# An integrative analysis revealing cuproptosis-related lncRNAs signature as a novel prognostic biomarker in hepatocellular carcinoma

**DOI:** 10.3389/fgene.2023.1056000

**Published:** 2023-02-10

**Authors:** Xilang Chen, Mengyu Sun, Weibo Feng, Jie Chen, Xiaoyu Ji, Meng Xie, Wenjie Huang, Xiaoping Chen, Bixiang Zhang, Yongzhan Nie, Daiming Fan, Kaichun Wu, Limin Xia

**Affiliations:** ^1^ State Key Laboratory of Cancer Biology, National Clinical Research Center for Digestive Diseases and Xijing Hospital of Digestive Diseases, Fourth Military Medical University, Xi’an, Shaanxi, China; ^2^ Hubei Key Laboratory of Hepato-Pancreato-Biliary Diseases, Department of Gastroenterology, Institute of Liver and Gastrointestinal Diseases, Tongji Hospital of Tongji Medical College, Huazhong University of Science and Technology, Wuhan, Hubei, China

**Keywords:** hepatocellular carcinoma, cuproptosis, lncRNAs, prognostic signature, immune infiltration

## Abstract

**Background:** Cuproptosis is a newly defined form of cell death, whether cuproptosis involved in hepatocellular carcinoma (HCC) remains elusive.

**Method:** We obtained patients’ RNA expression data and follow-up information from University of California Santa Cruz (UCSC) and The Cancer Genome Atlas (TCGA). We analyzed the mRNA level of Cuproptosis-related genes (CRGs) and performed univariate Cox analysis. Liver hepatocellular carcinoma (LIHC) was chosen for further investigation. Real-Time quantitative PCR (RT-qPCR), Western blotting (WB), Immunohistochemical (IHC), and Transwell assays were used to determine expression patterns and functions of CRGs in LIHC. Next, we identified CRGs-related lncRNAs (CRLs) and differentially expressed CRLs between HCC and normal cases. Univariate Cox analysis, least absolute shrinkage selection operator (LASSO) analysis and Cox regression analysis were used to construct the prognostic model. Univariate and multivariate Cox analysis was used to assess whether the risk model can act as an independent risk factor of overall survival duration. Different risk groups performed immune correlation analysis, tumor mutation burden (TMB), and Gene Set Enrichment Analysis (GSEA) analysis were performed in different risk groups. Finally, we assessed the performance of the predictive model in drug sensitivity.

**Results:** CRGs expression levels have significant differences between tumor and normal tissues. High expression of Dihydrolipoamide S-Acetyltransferase (*DLAT*) correlated to metastasis of HCC cells and indicated poor prognosis for HCC patients. Our prognostic model consisted of four cuproptosis-related lncRNA (AC011476.3, AC026412.3, NRAV, MKLN1-AS). The prognostic model performed well in predicting survival rates. The results from Cox regression analysis suggested that risk score can serve as an independent prognostic element for survival durations. Survival analysis revealed that low risk patients have extended survival periods compared with those with high risk. The results of the immune analysis indicated that risk score has a positive correlation with B cell and CD4^+^ T cell Th2, while has a negative relationship with endothelial cell and hematopoietic cells. Besides, immune checkpoint genes have higher expression folds in the high-risk set than in the low-risk set. The high-risk group had higher rates of genetic mutation than the low-risk set while having a shorter survival time. GSEA revealed the signaling pathways enriched in the high-risk group were mostly immune-related, while metabolic-related pathways were enriched in the low-risk group. Drugs sensitivity analysis indicated that our model has the ability to predict the efficacy of clinical treatment.

**Conclusion:** The Cuproptosis-related lncRNAs prognostic formula is a novel predictor of HCC patients’ prognosis and drug sensitivity.

## 1 Introduction

Primary liver cancer has a high incidence and death rate worldwide, which brought a huge burden on human health. The incidence rates and mortality rates of liver cancer were much higher in transitioned countries, especially in East Asia and Africa (Bray et al., 2018; Llovet et al., 2021). It is predicted that about one million persons will be diagnosed with liver cancer annually by 2025 (Llovet et al., 2021). Hepatocellular carcinoma (HCC), the dominant type of primary liver malignancies, is often found at the III-IV stage and thus has a disappointing prognosis (Forner et al., 2018). Currently, the efficacy of HCC treatment strategies is not satisfactory due to many aspects, such as high heterogeneity and drug resistance (Zhu et al., 2017). It is urgent for us to discover novel biomarkers, prognosis-related signature, and individualized treatment to extend HCC patients’ survival time and improve their life quality.

Copper-induced cell death (Cuproptosis) is distinct from all other already known regulated cell death forms, including four types, apoptosis, ferroptosis, pyroptosis, and necroptosis. In this type of cell death, mitochondrial metabolism level is the determining factor, lipoylated tricarboxylic acid enzymes increased in the tricarboxylic acid cycle active cells, copper directly combines with the lipoyl moiety and then aggregates lipoylated protein, lost Fe-S cluster–containing proteins, finally induced acute proteotoxic stress and cell death (Tsvetkov et al., 2022). Copper is like a double-edged sword, which exerts distinct roles when involved in different signaling pathways. For instance, copper promoted human breast cancer cells’ angiogenesis and progression *via* HIF-1α/GPER/VEGF signaling pathways (Rigiracciolo et al., 2015), while exerting suppressive roles to cervical cancer cells through PI3K/AKT/mTOR signaling pathway (Chen et al., 2021a). Previous studies reported that both copper ionophores and copper chelators have anti-cancer properties (Adsule et al., 2006; Li et al., 2020; Cui et al., 2021; Gou et al., 2021). Unfortunately, the functions of cuproptosis in HCC have not been identified and remain a black hole. Hence, cuproptosis may be a new target for HCC therapies, and it is imperative for us to unveil the underlying mechanisms and the potentiality for biomarkers associated with cuproptosis in HCC.

Long non-coding RNAs (lncRNAs) are special subtypes of RNA (Noh et al., 2018). Increasing evidence suggests that lncRNAs have vital roles in both physiological and pathological conditions (Fatica and Bozzoni, 2014; Morlando and Fatica, 2018). lncRNAs involved in multiple biological functions and disease statuses by regulating gene expression through various mechanisms (Peng et al., 2017). For example, recently published studies have shown that lncRNA-PNUTS activate the EMT pathway by targetting downstream effector Zinc Finger E-Box Binding Homeobox 1 (*ZEB1*) to increase HCC metastatic properties (Zhao et al., 2022). Accumulated evidence has shown the interaction between lncRNAs and regulated cell death-related genes play essential roles in HCC progression (Zhang et al., 2022a; Liu et al., 2022). However, whether cuproptosis-related (CR)-lncRNAs have roles in HCC and what roles CR-lncRNAs play are obscure, and their importance for acting as therapeutic targets and prognostic biomarkers in HCC needs to be deeply investigated.

In our work, we combined bioinformatic analysis and experimental methods to establish and validate a cuproptosis-related lncRNAs (CRLs) prognostic signature. We also developed a nomogram to predict the clinical outcomes of individual patients. The correlation between the risk scores and immune cells, immune checkpoint genes, and sensitivity of clinical treatment has also been explored. Finally, we also investigate the pathways enriched in different risk groups. In conclusion, CRLs prognostic signature can be a novel predictor of HCC patients’ prognosis and provide references to the therapeutic decision.

## 2 Materials and methods

### 2.1 Data collection

We collected information on 33 TCGA cancers and from the UCSC (http://genome.ucsc.edu), including RNA-Seq data, clinical information, survival data, and immune subtypes. RNA-Seq data were downloaded as Fragment Per Kilobase Million (FPKM) format. After downloading RNA-Seq, we transformed the FPKM format into TPMs (transcripts per kilobase million).

### 2.2 Retrieval of cuproptosis-related genes (CRGs) and pan-cancer analysis of CRG

We determined cuproptosis genes through screened previous literature (Tsvetkov et al., 2022). Then, we assessed the expression level of these CRGs in pan-cancer *via* “limma” R packages, and the differential expression of CRGs was visualized as boxplot and heat map. The correlation between CRGs was explored *via* “corrplot” packages. Univariate Cox regression analyses were performed by “survival” packages to acquire prognostic-related CRGs in pan-cancer, and the results were shown as forest maps. Besides, the expression level of CRGs in different immune subtypes was also been investigated.

### 2.3 Expression patterns and prognostic values of CRGs in HCC

We also assessed the expression levels of these CRGs in HCC *via* “limma” packages. The prognostic cuproptosis-related gene in HCC was identified *via* the Kaplan-Meier methods, and the best cutoff was set as criteria for dividing the whole cohort into high-expression cluster and low-expression cluster. Gene transfer format file was obtained from Ensembl2 to identify mRNA and lncRNA. The correlation between CRGs and all lncRNAs was analyzed, those with correlation coefficient >0.4, and *p*-value <0.05 were identified as cuproptosis-related lncRNAs (CRLs). To visualize the association between these lncRNAs and CRGs, we drew the co-expression network. Then, we picked differentially expressed lncRNAs between HCC and normal samples according to the standards: logFC ≥ 1, FDR ≤ 0.05.

### 2.4 Construction and verification of cuproptosis-related prognostic signature

A sum of 370 HCC patients was randomly allocated into training cohort and testing cohort. The previously determined differentially expressed CRLs were further selected by Univariate Cox analysis to choose prognosis-associated CRLs. Next, LASSO and Cox regression analyses were used to develop the predictive model. Lasso regression operated 1,000 cycles to acquire the CRLs with the smallest cross-validation error, and then Cox proportional hazards regression analysis and model construction was carried out. The formula for calculating cuproptosis-related prognostic risk scores of all HCC cases is as follows.

$$RiskScore=\overset{n}{\underset{i=1}{\sum }}Expi*Wi$$

Where Exp and W represent the express value of every lncRNA and the corresponding coefficient of each lncRNA, respectively. In the training cohorts, the total patients were divided into high-risk and low-risk groups based on the median risk scores. The overall survival (OS) time of the two groups was generated by Kaplan-Meier curve, and log rank test was used to compare OS between these two groups. The predictive ability of the risk model was assessed by receiver operating characteristic (ROC) curve, including the predictive accuracy of 1, 3, and 5-year survival rates. Finally, the risk model was validated in the testing set and total cases.

### 2.5 Comparison of cuproptosis-related prognostic signature and other cell death-related prognostic models

We calculated Harrell’s concordance index (C-index) by packages “survival” and “survcomp” to evaluate the concordance of our prognostic model. To figure out predictive variances between cuproptosis-related prognostic and other cell death-related prognostic models, we revalidated other models in TCGA databases, and the C-index of these models has also been calculated for comparison. Other cell death-related prognostic models include pyroptosis (Li et al., 2022; Deng et al., 2022), ferroptosis (Zhang et al., 2022a; Wang et al., 2021a), necroptosis (Yang and Jiang, 2022), and apoptosis (Zhu et al., 2020; Yan et al., 2021).

### 2.6 Construction of the nomogram model

Nomogram is a useful prediction tool that has been frequently used for cancer prognosis; it integrates various prognosis-related factors. Based on Cuproptosis-related genes score (CRGscore) and clinical characteristics, we developed a nomogram to estimate the outcomes of an individual case more accurately. We combined the univariate and multivariate Cox analysis to evaluate the relevancy between clinical variables, risk scores, and patients’ outcomes and selected the prognostic index and corresponding coefficient from the results of regression analysis. Then, the nomogram was developed by “survival” and “rms” packages and visualized by “regplot.” The consistency and accuracy of the nomogram model were evaluated by calibration analysis. ROC curve was carried out to evaluate the capability of the nomogram for predicting 1, 3, 5-year survival rates. Finally, we compared the predictive performance of multiple variables, and the results were presented in the ROC curve.

### 2.7 Immune analysis and tumor mutation burden (TMB)

ESTIMATE is a novel algorithm that uses transcriptional data of cancer to calculate three parameters to estimate the Infiltrating cells and tumor purity, including ImmuneScore, StromalScore, and ESTIMATEScore (Yoshihara et al., 2013). The proportion of immune-stromal components was conducted by the “ESTIMATE” packages *via* R software. The correlation of CRGs and above scores was evaluated by “corrplot” packages. The relationship between risk scores and immune cells in HCC was investigated by multiple widely used algorithms, including XCELL, TIMER, QUANTISEQ, MCPCOUNTER, EPIC, CIBERSORT-ABS, and CIBERSORT. The expression level of immune checkpoint (ICP) genes and TMB status has been assessed between the two groups. We investigated the relationship between survival periods and TMB status and combined TMB status and risk scores to operate stratified analyses.

### 2.8 Gene set enrichment analysis

To investigate whether there are molecular function and biological differences in different risk groups, GSEA analysis with Gene Ontology (GO) and Kyoto Encyclopedia of Genes and Genomes (KEGG) gene sets was performed by “clusterProfiler” packages.

### 2.9 Drug sensitivity prediction

To determine whether the risk model has guiding values in clinical decision-making, we computed half-inhibitory concentrations (IC50) of anti-cancer drugs obtained from TCGA datasets. The drug sensitivity prediction was performed *via* “pRRophetic” packages.

A detailed description of the methods used in this study were listed in the Supplementary Material.

## 3 Results

### 3.1 Screening of CRGs and expression patterns analysis

**FIGURE 1:**
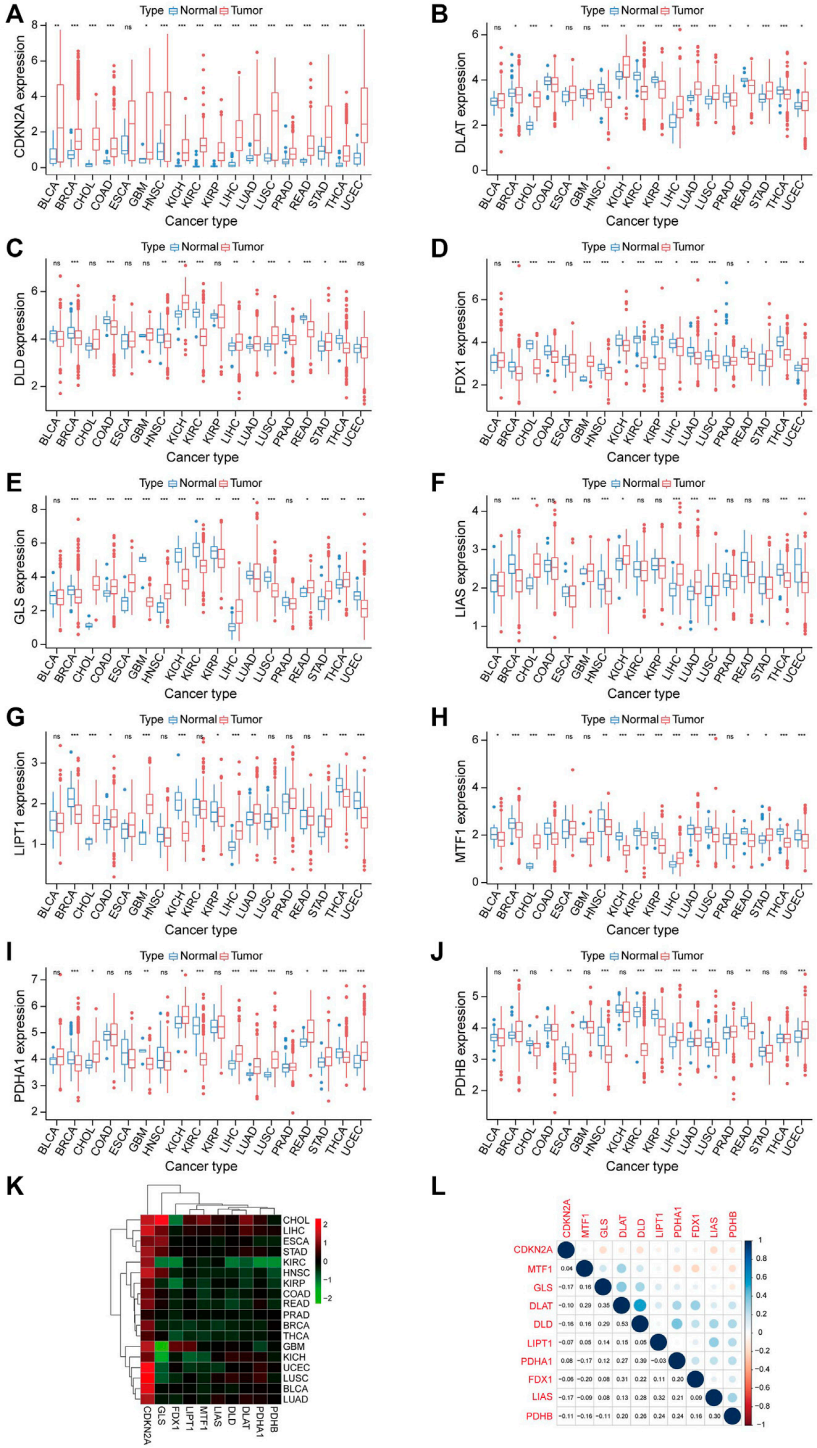
Expression analysis of CRGs in Pan-cancer. **(A–J)** Shows the expression level of 10 CRGs in cancer and normal tissues. Tumor labeled with red color, normal tissue labeled with blue color. *p < 0.05, **p < 0.01, ***p < 0.001. (**K)** The relationship between cancer and the expression level of 10 CRGs is also represented in the heatmap. **(L)** Correlation between 10 CRGs.

Supplementary Figure S1 shows the workflow of this study. To determine the gene involved in the biological process of cuproptosis, we searched previously published literatures (Tsvetkov et al., 2022). A total of 10 genes were identified, including Ferredoxin 1 (*FDX1*), Lipoic Acid Synthetase (*LIAS*), Lipoyltransferase 1 (*LIPT1*), Dihydrolipoamide Dehydrogenase (*DLD*), Dihydrolipoamide S-Acetyltransferase (*DLAT*), Pyruvate Dehydrogenase E1 Subunit Alpha 1 (*PDHA1*), Pyruvate Dehydrogenase E1 Subunit Beta (*PDHB*), Metal Regulatory Transcription Factor 1 (*MTF1*), Glutaminase (*GLS*), and Cyclin Dependent Kinase Inhibitor 2A (*CDKN2A*). Next, we carried out a pan-cancer analysis of these ten genes. The expression level of CRGs is significantly different between cancer cases and healthy control (Figures 1A–J). Among these CRGs, *CDKN2A* is outstanding for it has high expression in almost all cancer except for esophageal carcinoma (ESCA), while the remaining nine genes have distinct expression patterns in specific cancer types. For instance, *PDHB* is higher in breast invasive carcinoma (BRCA), LIHC, lung adenocarcinoma (LUAD), and uterine corpus endometrial carcinoma (UCEC) than in their corresponding normal tissues while lower in colon adenocarcinoma (COAD), ESCA, head and neck squamous cell carcinoma (HNSC), kidney renal clear cell carcinoma (KIRC), kidney renal papillary cell carcinoma (KIRP), lung squamous cell carcinoma (LUSC), and rectum adenocarcinoma (READ) than in paired normal samples. Heatmap also confirmed the CRGs correlated with TCGA cancer. Among ten genes, *GLS* has the highest positive and negative correlation with cholangiocarcinoma (CHOL) and glioblastoma (GBM), respectively (Figure 1K). We also performed co-expression analysis in pan-cancer, Figure 1L showed the correlation of CRGs. *DLD* and *DLAT* have the highest correlation in pan-cancer, followed by *DLD* and *PDHA1*.

### 3.2 Prognostic value of CRGs in TCGA cancers

**FIGURE 2:**
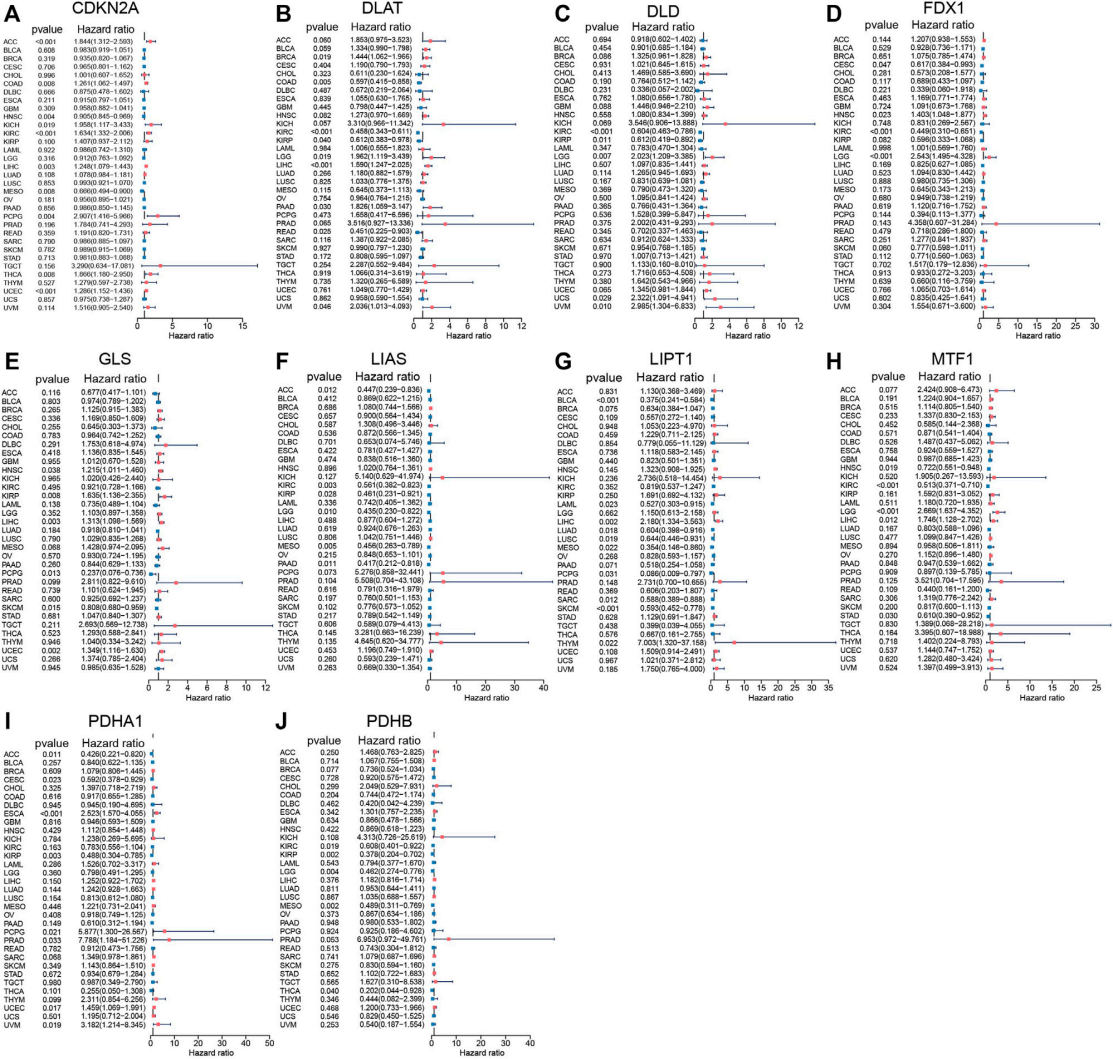
Prognostic values of 10 CRGs in pan-cancer explored by Univariate Cox analysis. **(A)** *CDKN2A*. **(B)** *DLAT*. **(C)** *DLD*. **(D)** *FDX1*. **(E)** *GLS*. **(F)** *LIAS*. **(G)** *LIPT1*. **(H)** *MTF1*. **(I)** *PDHA1*. **(J)** *PDHB*.

Univariate Cox regression analyses were performed based on TCGA data to explore the prognostic value of CRGs in pan-cancer. The results indicated that CRGs have a tight relationship with the outcomes of most cancer (Figures 2A–J). For example, *CDKN2A* correlated with the most cancer (10/33), and its high expression correlated with worse outcome in adrenocortical carcinoma (ACC) (*p* < 0.001, HR = 1.844), COAD (*p* = 0.008, HR = 1.261), kidney chromophobe (KICH) (*p* = 0.019, HR = 1.958), KIRC (*p* < 0.001, HR = 1.634), LIHC (*p* = 0.003, HR = 1.248), pheochromocytoma and paraganglioma (PCPG) (*p* = 0.004, HR = 2.907), thyroid carcinoma (THCA) (*p* = 0.008, HR = 1.866), UCEC (*p* < 0.001, HR = 1.286), while predicted better outcome in HNSC (*p* = 0.004, HR = 0.905), mesothelioma (MESO) (*p* = 0.008, HR = 0.666). Cox regression analysis showed 6 of 10 CRGs correlated with the prognosis of LIHC; we speculated that CRGs might play a vital role in the progression of LIHC.

### 3.3 Expression patterns and prognostic values of CRGs in LIHC

**FIGURE 3:**
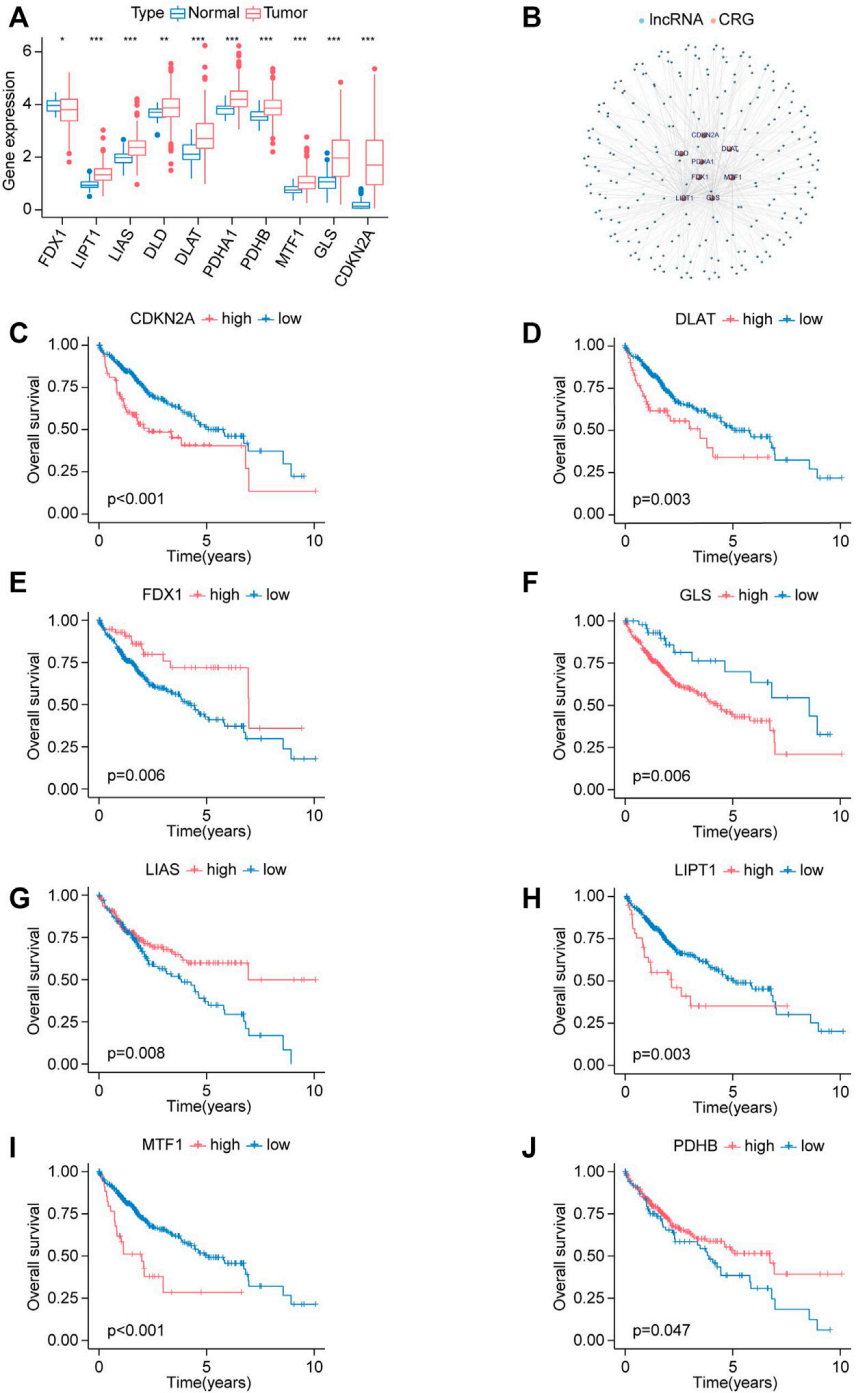
Expression signature and prognostic analysis of CRGs in HCC. **(A)** The expression status of CRGs in HCC presented in box plot. Tumor labeled with red color, normal tissue labeled with blue color. *p < 0.05, **p < 0.01, ***p < 0.001. **(B)** Co-expression network of lncRNAs and cuproptosis-related genes. **(C–J)** The survival curves for high-expression and low-expression groups are classified by the expression levels of CRGs. **(C)** *CDKN2A*. **(D)** *DLAT*. **(E)** *FDX1*. **(F)** *GLS*. **(G)** *LIAS*. **(H)** *LIPT1*. **(I)** *MTF1*. **(J)** *PDHB*.

Next, LIHC has been chosen for further investigation. We explored the expression status of CRGs in HCC, and the results indicated that most genes were upregulated in HCC compared to normal tissues except for *FDX1*, including *LIAS*, *LIPT1*, *DLD*, *DLAT*, *PDHA1*, *PDHB*, *MTF1*, *GLS*, *CDKN2A* (Figure 3A).

Kaplan-Meier analysis was applied to illustrate whether CRGs have prognostic values in HCC. Elevated expression of *CDKN2A*, *DLAT*, *GLS*, *LIPT1*, and *MTF1* was correlated with shorter OS in HCC, while upregulation of *FDX1*, *LIAS*, *PDHB* has longer OS in HCC (Figures 3C–J).

lncRNA-CRGs co-expression network was constructed to explore the relationship between these 10 CRGs and all lncRNA (Figure 3B). A total of 221 lncRNAs were identified according to the selected criteria.

### 3.4 RT-qPCR analysis of the CRGs

**FIGURE 4:**
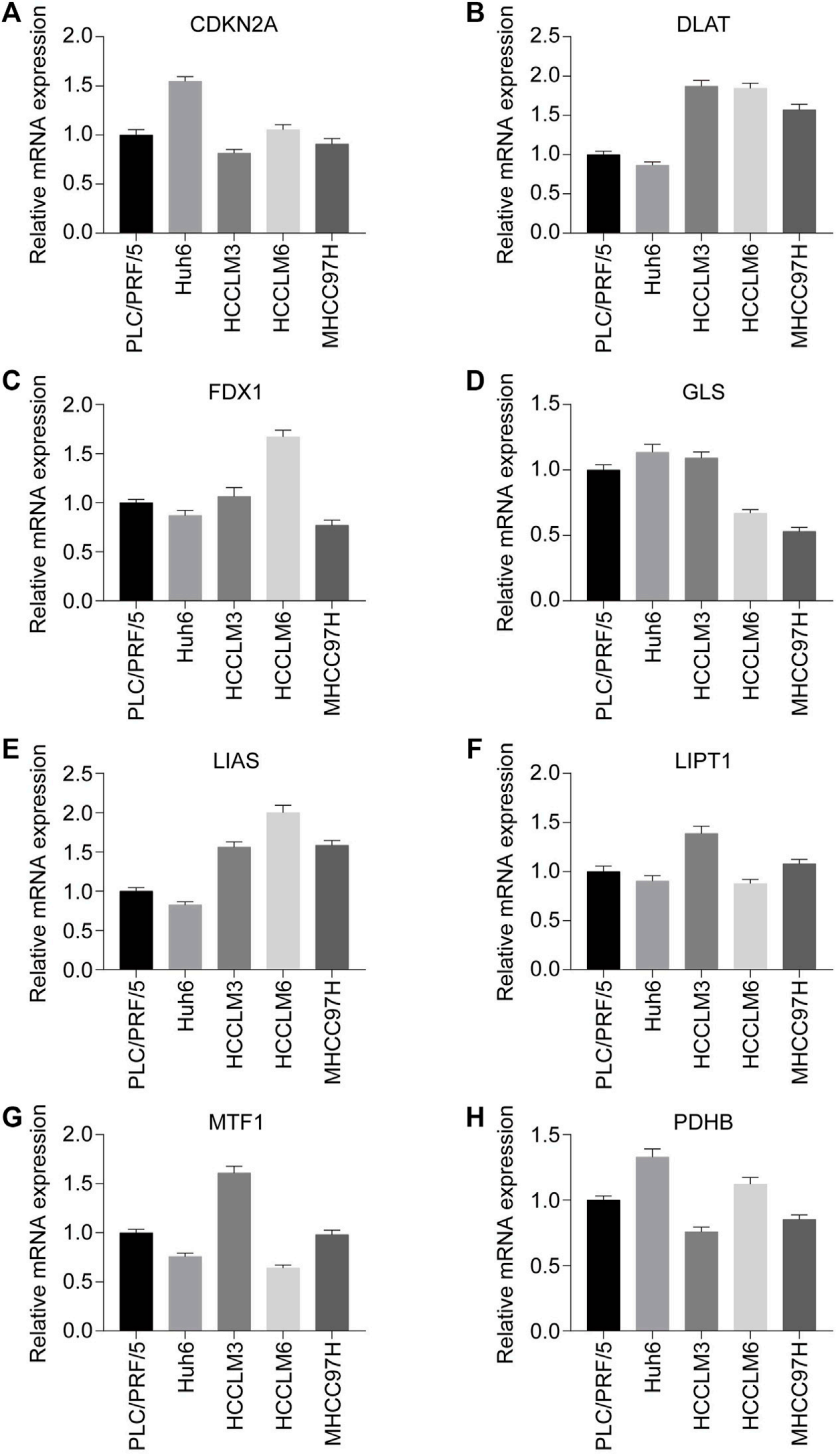
Expression status of CRGs with prognostic values in HCC cell lines (PLC/PRF/5, Huh6, HCCLM3, HCCLM6, MHCC97H) were assessed by RT-qPCR. **(A)** *CDKN2A*. **(B)** *DLAT*. **(C)** *FDX1*. **(D)** *GLS*. **(E)** *LIAS*. **(F)** *LIPT1*. **(G)** *MTF1*. **(H)** *PDHB*. *p < 0.05, **p < 0.01, ***p < 0.001.

To explore the expression pattern of these eight prognosis-related CRGs in the cell level, we performed qPCR in five HCC cell lines. As shown in Figure 4, high metastatic capabilities cell (HCCLM3, HCCLM6, MHCC97H) has higher expression level of *DLAT*, *LIAS* when compared with low metastatic capabilities cell (PLC/PRF/5, Huh6), while other genes have no significant differences. This result indicated *DLAT* and *LIAS* may play a pro-metastasis role in HCC and therefore influence the outcome of HCC patients.

### 3.5 Distinct expression level of *DLAT* in different samples

**FIGURE 5:**
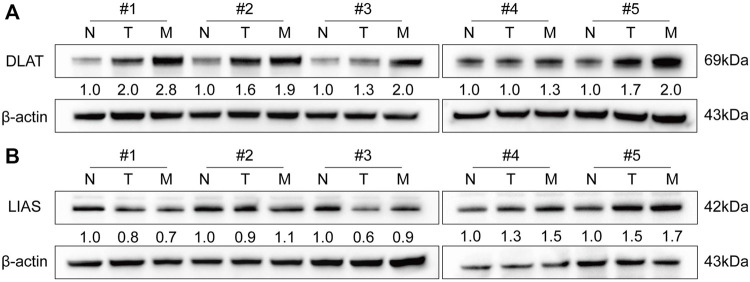
The expression signature of DLAT and LIAS in five pairs of HCC and adjacent non-tumor samples and five metastatic HCC specimens. **(A)** DLAT. **(B)** LIAS.

To determine the functions of *DLAT* and *LIAS* in HCC, we performed WB to quantify the protein level of *DLAT* and *LIAS* in five pairs of adjacent non-tumor, primary HCC, and five metastatic HCC samples. The result showed metastatic HCC has a higher expression level of *DLAT* than primary HCC, and primary HCC has higher expression level of *DLAT* than adjacent non-tumor (Figure 5A). While *LIAS* has no such significant trends (Figure 5B). The result of WB from clinical samples illustrated that *DLAT* has distinct expression levels in normal tissues, primary HCC, and metastatic HCC. Based on this finding, we inferred that high expression level of *DLAT* can promote HCC metastasis.

### 3.6 *DLAT* is upregulated in HCC tissues and indicates poor outcomes

**FIGURE 6:**
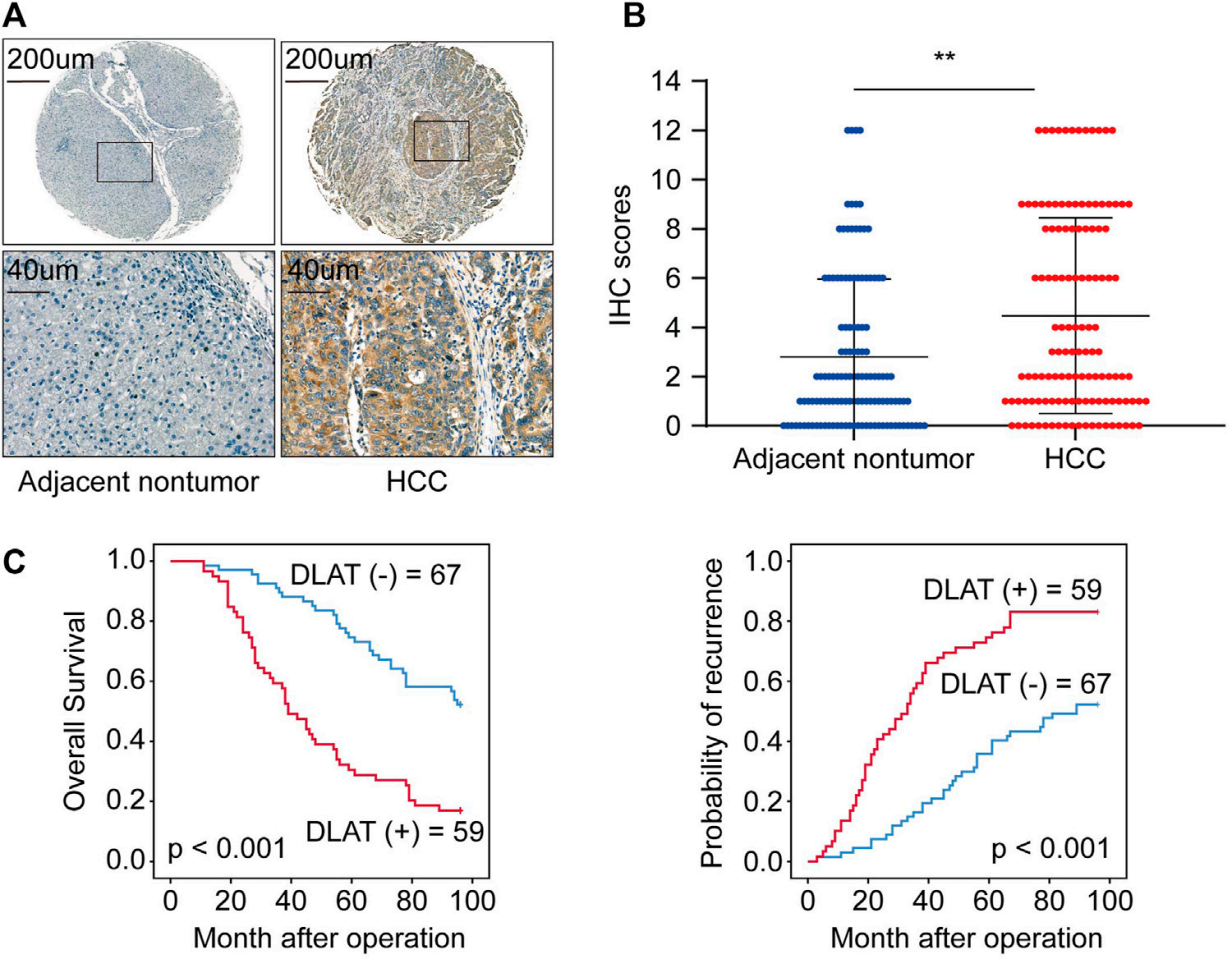
DLAT is upregulated in HCC tissues and indicates poor outcome in HCC. **(A)** The representative IHC staining of DLAT in non-tumor (negative) and HCC (positive). **(B)** The chart shows IHC scores of DLAT expression in 126 pairs of HCC and normal tissues. **(C)** Survival analysis suggests high expression level of DLAT indicated poor OS and high recurrence rates in HCC.

We used IHC to determine the protein level of *DLAT* with a tissue array of 126 HCC patient samples and performed Kaplan-Meier analysis and Cox regression to uncover the prognostic significances of DLAT. The IHC result showed the expression of DLAT was significantly higher in HCC than in their corresponding adjacent non-tumor tissues (Figures 6A, B). K-M survival analysis showed high DLAT expression group had worse OS and higher recurrence probability than low expression group (Figure 6C). Correlation analysis showed DLAT has positive correlation with tumor encapsulation, poor differentiation, microvascular invasion, and higher Tumor Node Metastasis (TNM) stage (Table 1). These results illustrated upregulation of DLAT can promote HCC progression, and the expression of DLAT can predict HCC patients’ clinical outcomes.

**TABLE 1:** Correlation between DLAT expression and clinicopathological characteristics of HCCs in cohorts of human HCC tissues.

Clinicopathological variables		Tumor DLAT expression		*p*-value
		Negative (*n* = 67)	Positive (*n* = 59)	
Age		52.60 (11.07)	49.07 (13.95)	0.116
Sex	Female	17	10	0.250
	Male	50	49	
Serum AFP	≤20 ng/mL	20	12	0.221
	>20 ng/mL	47	47	
Virus infection	HBV	26	16	0.200
	HCV	8	7	
	HBV + HCV	1	5	
	None	32	31	
Cirrrhosis	Absent	14	14	0.703
	Present	53	45	
Child-pugh score	Class A	47	47	0.221
	Class B	20	12	
Tumor number	Single	47	20	<0.001
	Multiple	20	39	
Maximal tumor size	≤5 cm	31	29	0.746
	>5 cm	36	30	
Tumor encapsulation	Absent	18	30	0.006
	Present	49	29	
Microvascular invasion	Absent	49	31	0.017
	Present	18	28	
Tumor differentiation	I-II	44	21	0.001
	III-IV	23	38	
TNM stage	I-II	57	39	0.013
	III-IV	10	20	

### 3.7 Overexpression of DLAT enhances HCC metastatic property

**FIGURE 7:**
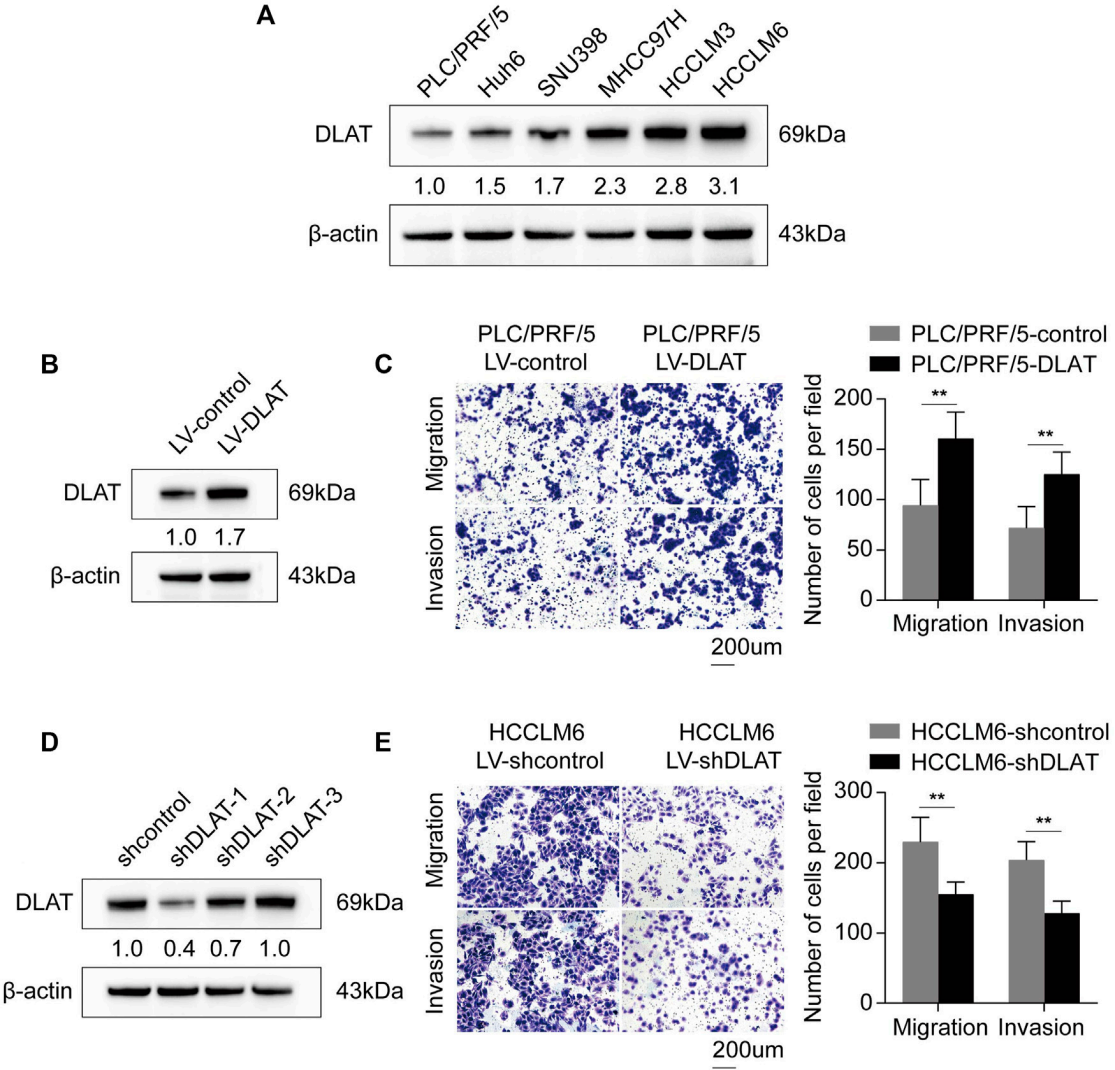
Upregulated DLAT enhances HCC cell migration and invasion capacities. **(A)** The expression status of DLAT in HCC cell lines. **(B)** The result of Western blotting confirmed that DLAT was upregulated in PLC/PRF/5 treated with DLAT-overexpressing lentivector. **(C)** Overexpression of DLAT promoted migration and invasion abilities of PLC/PRF/5. **(D)** The result of western blotting confirmed that DLAT was significantly knocked down in HCCLM6. **(E)** Downregulation of DLAT in HCCLM6 inhibited migration and invasion abilities of HCCLM6.

We performed transwell assays to investigate whether the differential expression of DLAT have influence on metastatic properties of HCC cells. Firstly, we determined the expression patterns of DLAT in HCC cells. The result showed DLAT was upregulated in high metastatic cells compared with low metastatic cell (Figure 7A). Next, PLC/PRF/5 was used to construct a stable DLAT overexpressing cell (PLC/PRF/5-DLAT) (Figure 7B), and the expression of DLAT in MHCC97H was knocked down (MHCC97H-shDLAT) (Figure 7D). Transwell assays showed upregulation of DLAT in low metastatic capability cells increased the migration and invasion ability, while downregulation of DLAT in high metastatic capability cells has the opposite effect (Figures 7C, E).

### 3.8 Construction and verification of CR-lncRNA prognostic signature

370 HCC patients were included in this analysis, the total HCC cohorts were randomly separated into two groups: the training group (185 cases) and the testing group (185 cases). Among 221 CR-lncRNAs, 174 lncRNAs were labeled differentially expressed CR-lncRNAs (DECR-lncRNA) based on the criteria: FDR <0.05, | log2FC| > 1 (Supplementary Figure S2A). Then, univariate cox analysis was applied to these DECR-lncRNA in the training group for identified prognostic-related lncRNA. A total of 29 lncRNAs were considered as having prognostic values (Supplementary Figure S2B). LASSO regression and Cox proportional hazard analyses were performed to determine the risk model (Supplementary Figures S2C, D). Finally, four lncRNAs were contained in the risk score model, including AC011476.3, Negative Regulator of Antiviral Response (NRAV), AC026412.3, and Muskelin 1-Antisense RNA (MKLN1-AS).

RiskScore=−3.158*AC011476.3+0.586* NRAV+2.708* AC026412.3+1.372* MKLN1−AS

**FIGURE 8:**
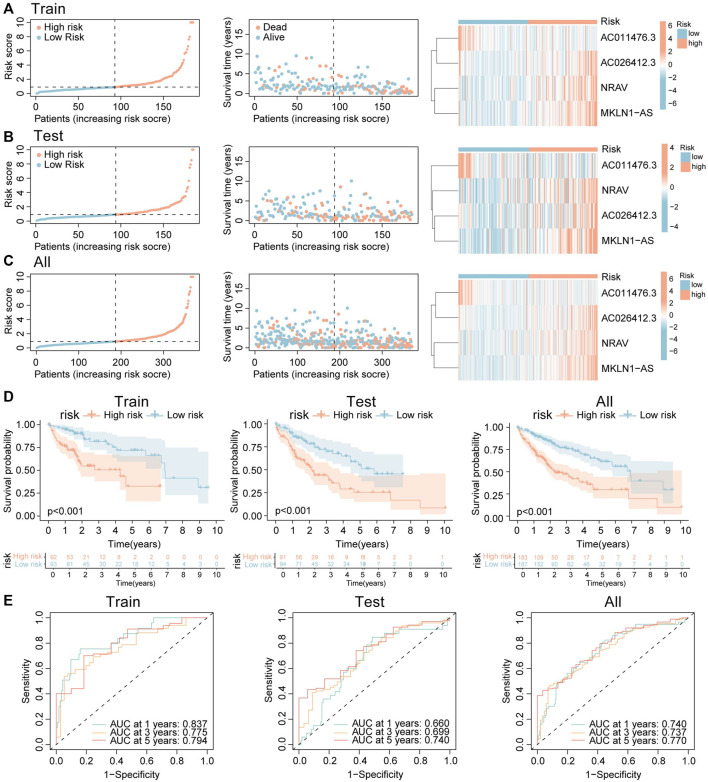
Construction and validation of the CRLs signature. **(A–C)** The distribution of risk score, survival status, and heatmap of the expression signature of four cuproptosis-related lncRNAs in **(A)** training cohort. **(B)** Testing cohort. **(C)** Overall cohort. **(D)** The K-M curves show HCC patients’ survival time and survival status in training, testing, and overall groups. **(E)** ROC curves show the efficacy of cuproptosis-related lncRNAs signature in forecasting overall survival time of 1, 3, 5 years in training, testing, and overall groups.

Then, each case was calculated with the risk score based on the above formula, and the training group was divided into two groups (high-risk and low-risk) according to the median risk value (0.904). The heatmap indicated that NRAV, AC026412.3, and MKLN1-AS have high expression trends in the high-risk group, while AC011476.3 showed the opposite trends (Figure 8A right). And the death rates increased as the risk scores rose (Figure 8A left and middle). Survival analysis was also performed in the training cohort to investigate the predictive role of the risk score in a patient’s prognosis (Figure 8D left). The results showed low-risk group has longer survival durations than the high-risk group (*p* < 0.001). Receiver operating characteristic (ROC) curve was constructed to assess the predictive ability of the risk model, and the ROC values of 1, 3, and 5-survival rates were 0.837, 0.775, and 0.794, respectively (Figure 8E left).

CR-lncRNA prognostic model has been validated in the testing set and whole groups. The expression patterns of these four lncRNAs and the distributed difference in mortality between different risk groups in the testing cohort and entire cohorts were akin to those of the training group (Figures 8B, C). In the testing cohort and entire cohorts, the results of K-M survival analysis are also consistent with that of the training cohort (Figure 8D middle and right). The area under curve (AUC) values 1, 3, and 5-survival rates in the testing cohort were 0.660, 0.699, 0.740, respectively, and in the entire cohort were 0.740, 0.737, 0.770, respectively (Figure 8E middle and right). The above results indicated that CR-lncRNA prognostic model has good performance in accuracy.

### 3.9 Validation of the expression and prognostic values of lncRNA

**FIGURE 9:**
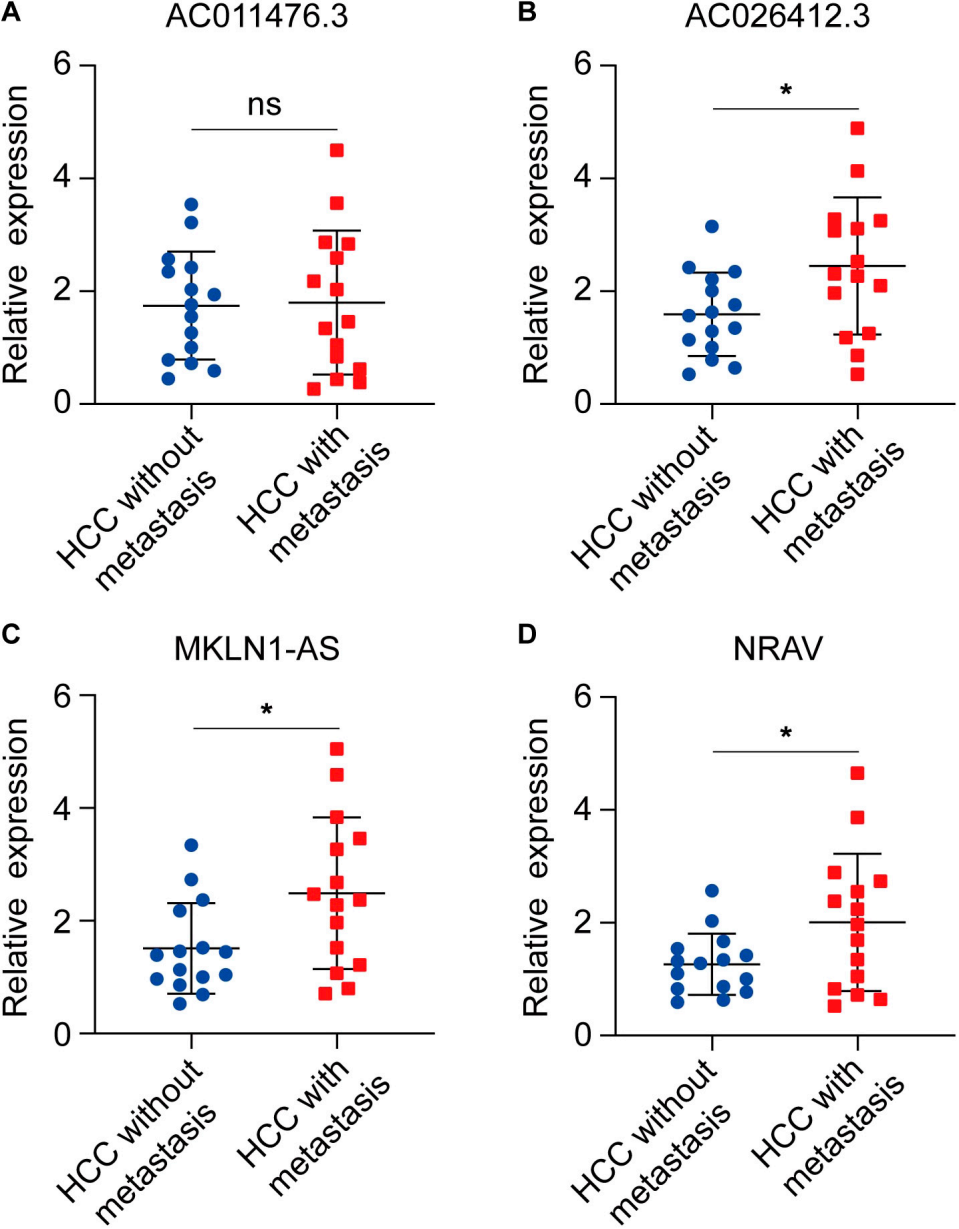
The expression status of four CRLs in primary HCC and metastatic HCC. **(A)** AC011476.3. **(B)** AC026412.3. **(C)** MKLN1-AS. **(D)** NRAV.

To explore whether the four CRGs-related lncRNA was correlated to the progression of HCC, we detected the expression level of AC011476.3, NRAV, AC026412.3, and MKLN1-AS in the clinical HCC sample. The results of qRT-PCR indicated that NRAV, AC026412.3, and MKLN1-AS are higher in metastatic HCC than primary HCC, while AC011476.3 showed no differences between the two distinct kinds of HCC group (Figures 9A–D **)**. The prognostic value of four lncRNA was further investigated by UALCAN online tool. Kaplan-Meier analysis showed high expression of NRAV, AC026412.3, and MKLN1-AS was significantly correlated to poor prognosis of HCC patients, while AC011476.3 has no significant prognostic values (Supplementary Figure S3).

### 3.10 Comparison of cuproptosis-related prognostic signature and other cell death-related prognostic models

**FIGURE 10:**
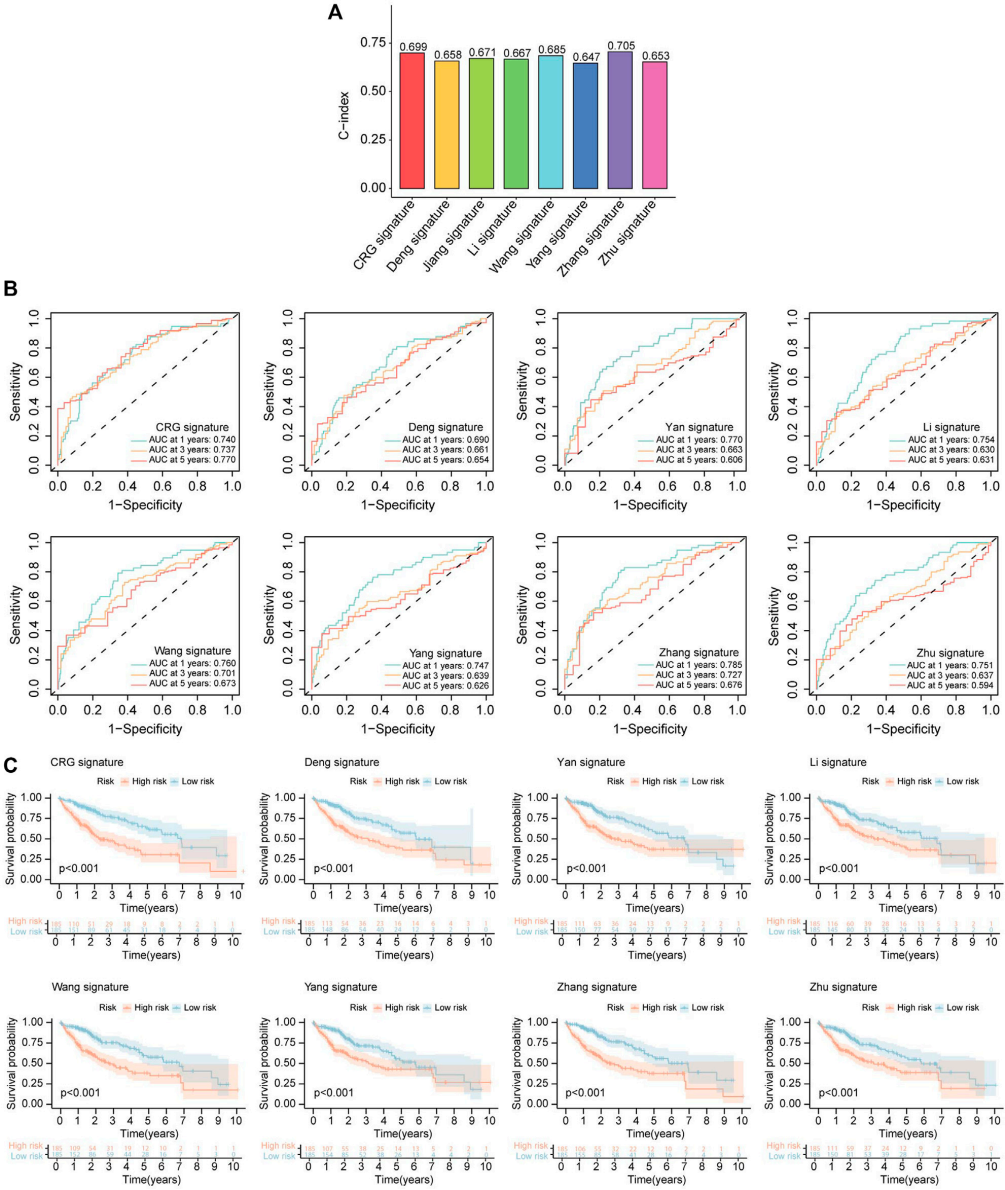
Comparison of CRLs prognostic signature and other models. **(A)** The C-index of our signature and other models. The C-index reflects the concordance of predicted results and actual situations. **(B)** ROC curves show the efficacy of cuproptosis-related lncRNAs signature and that of other models in forecasting overall survival time of 1, 3, 5 years. **(C)** The K-M curves show HCC patients’ survival time and survival status in CRLs signature and that of other models.

To further confirmed the good predictive properties of cuproptosis-related prognostic signature, we compared our model with other published cell death-related prognostic models. We revalidated all selected models based on the TCGA database. C-index of all interested models was calculated, and the results showed C-index of CR prognostic signature was better than most other models (Figure 10A). ROC curve and K-M analysis were also performed to compare the efficacy of the CR prognostic signature and other published works, and also confirmed that CR prognostic model was superior to other published signatures (Figures 10B, C).

### 3.11 Construction of the nomogram model

**FIGURE 11:**
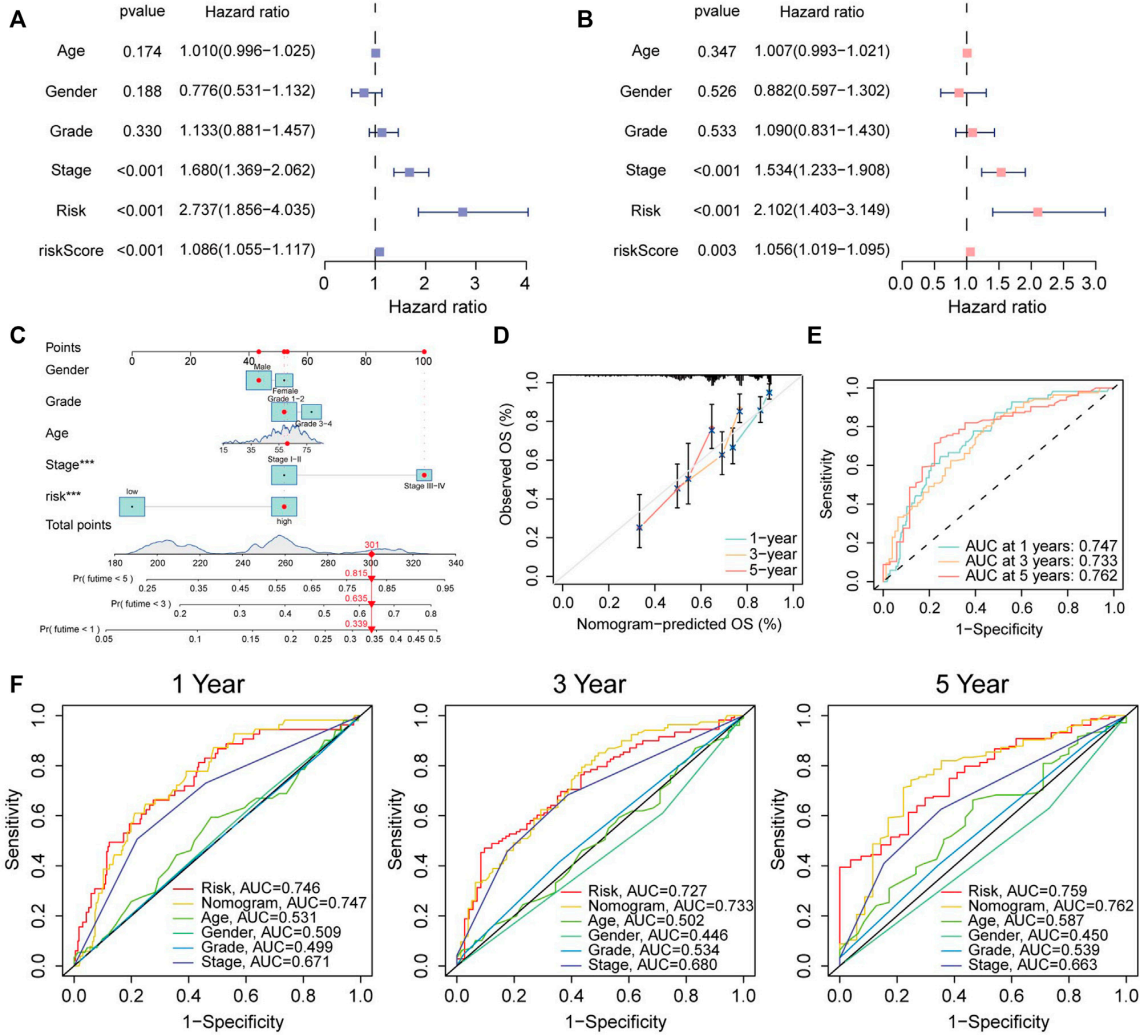
Construction and evaluation of nomogram. **(A)** Stage, risk, and risk score were considered significant. **(B)** Multivariate Cox regression analysis showed stage, risk, and risk score were independent prognostic predictors. **(C)** 1, 3, 5-year survival rates predicted by Nomogram. **(D)** The predictive abilities of nomograms in 1, 3, and 5 years of survival were evaluated by calibration. **(E)** ROC curves reflect the accuracy of OS predicted by nomogram. **(F)** The AUCs value of CRLs model and clinical characteristics for predicting 1, 3, 5 years survival. *p < 0.05, **p < 0.01, ***p < 0.001.

To assess the potentiality for risk score acting as prognostic determinant independently, we used Univariate and Multivariate Cox analyses to analyze the correlation between OS and risk score, and other clinical elements including age, gender, grade, and stage in the entire HCC cohort. The results from univariate cox analysis revealed that stage (*p* < 0.001), risk (*p* < 0.001), risk score (*p* < 0.001) was correlated with prognosis (Figure 11A). And the results of Multivariate Cox indicated that stage (*p* < 0.001), risk (*p* < 0.001), risk score (*p* < 0.003) can serve as independent risk factor for HCC patients (Figure 11B). The results also illustrated that risk stage rather than risk score have the nice performance to predict patient’s outcome [Risk: *p* < 0.001, HR = 2.737, confidence interval (CI) = 1.856–4.035, *p* < 0.001, HR = 2.102, CI = 1.403–3.149, for Uni-Cox and Multi-Cox, respectively].

Five parameters were used to construct the nomogram, including gender, grade, age, stage, and risk (Figure 11C). The predictive 1, 3, 5-survival rate was close to the actual observation (Figure 11D). The ROC curve was also plotted to assess the efficacy of the nomogram. The AUC value of nomogram at 1, 3, 5-survival rate was 0.747, 0.733, 0.762, respectively (Figure 11E).

Besides, the ROC curve was constructed by integrating multiple parameters to compare the efficacy of each indicator more specifically. Nomogram was outstanding in all included indicators (Figure 11F).

### 3.12 Immune analysis and TMB

**FIGURE 12:**
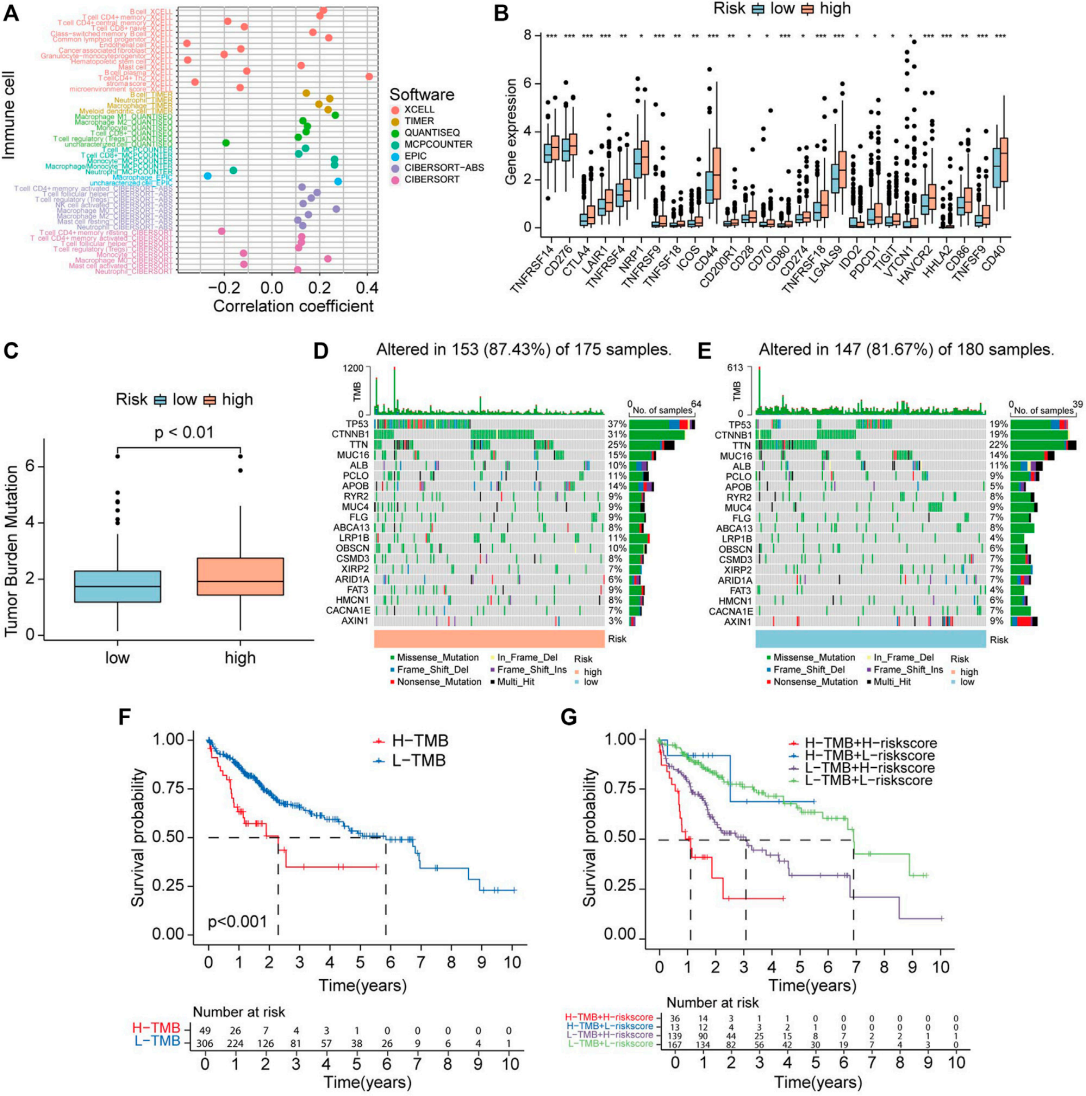
Correlation between CRLs score and immune infiltrating cells and tumor mutation burden (TMB). **(A)** Lollipop plot shows the relationship between CRLs score and immune cells analyzed by various algorithm. **(B)** The expression level of immune checkpoint genes in high-risk and low-risk groups. **(C)** TMB status in high-risk and low-risk groups. **(D)** OncoPrint showed the mutations status in the high CRLs score group. **(E)** OncoPrint showed the mutations status in the low CRLs score group. **(F)** The K-M curves show survival status and survival time in high-TMB and low-TMB groups. **(G)** Stratified survival analysis, including TMB and CRLs scores.

Supplementary Figure S4 showed the CRGs have distinct expression patterns in the immune subtype (C1-C6). Risk score has a positive correlation with B cells, common lymphoid progenitor, CD4^+^ T cell Th2, and has a negative relationship with endothelial cells and hematopoietic cells in HCC (Figure 12A). Supplementary Figure S5 represents the correlation of 10 CRGs and ESTIMATEScore, ImmuneScore, StromalScore, and TumorPurity in TCGA cancers. Immune checkpoint inhibitors (ICIs) play vital roles in the process of anti-HCC, we further analyzed the expression level of immune checkpoint (ICP) genes in different risk groups. Interestingly, all already recognized ICP genes were remarkably overexpressed in the high-risk groups (Figure 12B). This finding means HCC patients that have a high-risk score of CR prognostic may benefit more from ICIs therapy.

Accumulated gene mutation led to carcinogenesis. First, we analyzed the TMB level between two groups divided by the risk model. The results revealed that TMB levels in high-risk group are higher than in low-risk group (Figure 12C). To explore the difference in somatic mutations between CRGscore-risk groups, we analyzed the mutated gene in each group. *TP53* (37%), *CTNNB1* (31%), *TTN* (25%), *MUC16* (15%), and *APOB* (14%) were the top five genes that have the highest mutation rates in the high-risk group. While *TTN* (22%), *TP53* (19%), *CTNNB1* (19%), *MUC16* (14%), and *ALB* (11%) were the top five genes that have the highest mutation frequencies in low-risk group (Figures 12D, E).

Survival analysis indicated that high TMB level group has shorter OS than low TMB group (*p* < 0.001) (Figure 12F). Stratified survival analyses indicated that HCC patients identified with low risk have a much longer survival period compared with those labeled with high risk in the high-TMB group. Consistently, HCC patients identified with low-risk have extended OS compared with those identified with high-risk in the low-TMB group (Figure 12G). These findings suggest that combining TMB and risk score to predict the outcome of HCC may be more precise than TMB or risk score alone.

### 3.13 GSEA

**FIGURE 13:**
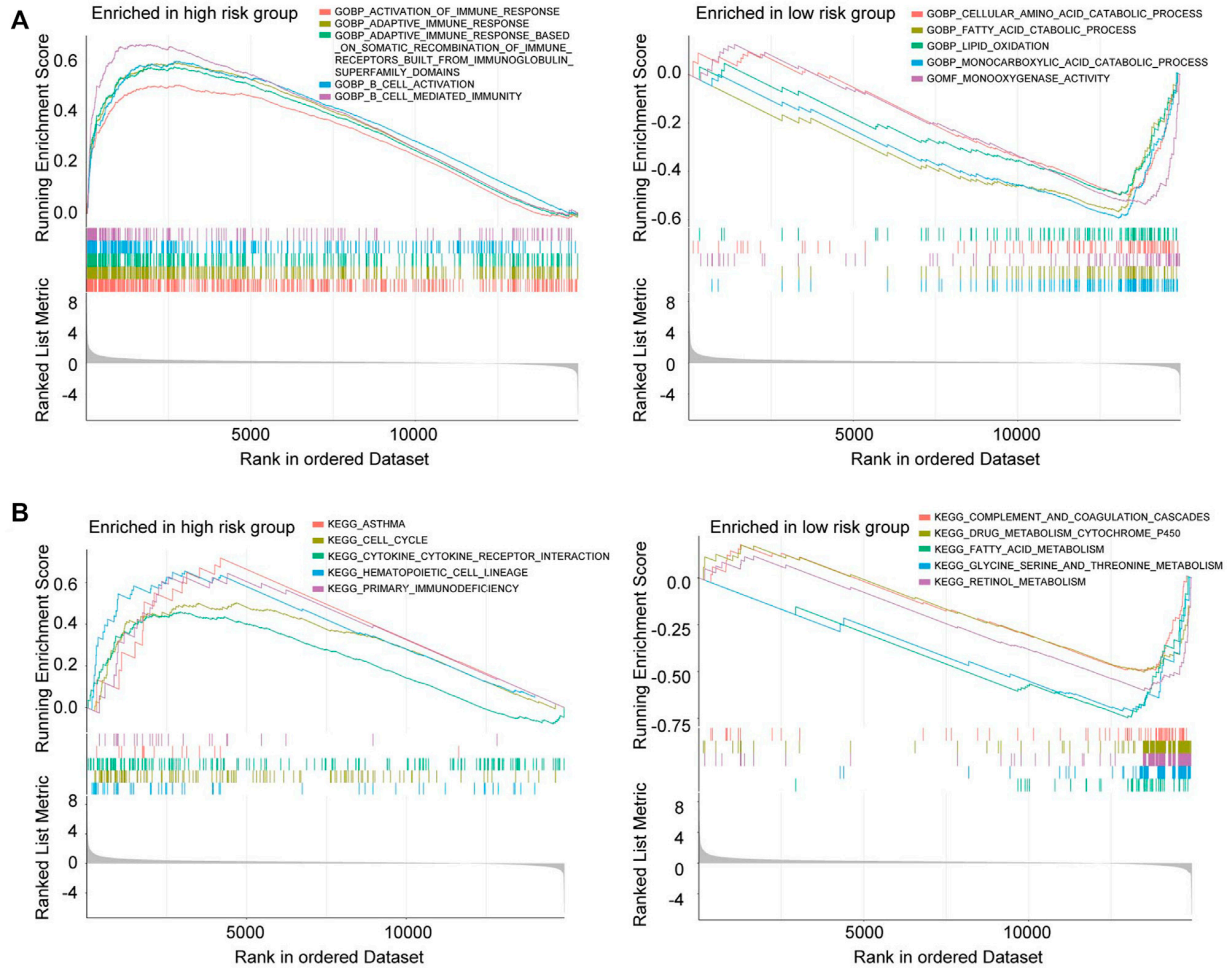
Gene Set Enrichment Analysis (GSEA) in the high-risk group and low-risk group. **(A)** GO enrichment analysis of high-risk group (left) and low-risk group (right). **(B)** KEGG enrichment analysis of high-risk group (left) and low-risk group (right).

GSEA was performed to investigate whether there are discrepancies between distinct CRGscore-risk groups in biological functions. The results indicated that many immune-related biological functions were enriched in high-risk group, such as B cell activation, B cell-mediated immunity, and adaptive immune response (Figure 13A left). Nevertheless, many metabolic functions were concentrated in low-risk group, like fatty acid catabolic process, lipid oxidation, and monocarboxylic acid catabolic process (Figure 13A right). Consistently, various immune-related pathways and metabolic pathways were enriched in high-risk and low-risk groups, respectively (Figure 13B).

### 3.14 Correlation between CRG signature and drugs sensitivity

IC_50_ was calculated to assess the sensitivity to various drugs. The IC_50_ values of Mitomycin. C, Midostaurin, Gemcitabine, Epothilone. B in high-risk group was significantly lower than low-risk group, which means HCC patients with higher risk scores benefit more from these drugs. On the contrary, the IC_50_ values of Bosutinib, Cyclopamine, Erlotinib, Nilotinib, and Temsirolimus in high-risk group were significantly higher than low-risk group, which indicated that HCC patients who have low-risk scores might benefit more from these drugs (Supplementary Figures S6, S7).

## 4 Discussion

HCC has higher mortality and recurrence rates than other malignancy tumors and imposes a huge burden on human health (Qiu et al., 2022). Although the application of novel agents significantly changed the treatment patterns of HCC, there are just minorities of HCC patients who showed great efficacy to these new strategies or obtained longstanding disease remission (Finn et al., 2020; Vogel et al., 2021). Thus, it is imperative for us to find reliable biomarkers in decision-making and select the patients who have the great potential to benefit from conventional treatment or novel therapies (Teufel et al., 2019).

lncRNAs have a vital role in cell differentiation, cell growth, and development, aberrant expression of lncRNAs correlated to various kinds of diseases, especially malignant tumor (Esteller, 2011; Peng et al., 2017). Increasing evidence indicated that lncRNAs are associated with HCC. Xu et al. (2018) demonstrated overexpression of lncRNA SNHG16 suppressed HCC growth and chemoresistance. LncRNA MFI2-AS1 plays a positive role in HCC progression and distant metastasis *via* upregulating the expression level of FOXM1 (Wei et al., 2020). Accumulating research or bioinformatic analysis identified lncRNAs can influence the phenotype of HCC by interacting with cell death, including pyroptosis, ferroptosis, and apoptosis (Mao et al., 2018; Zhang et al., 2022b). However, there are neither identified cuproptosis-related lncRNA nor research about cuproptosis-related lncRNA in HCC. Whether lncRNA has connections with cuproptosis or CRLs play roles in HCC remains confusing.

Copper is a vital nutrient for all mammals, though its concentrations are very low in serum. It links to critical biological processes, including energy metabolism, enzyme activity, and cell signal transduction (Ruiz et al., 2021). The concentrations of copper in the body are finely regulated by homeostatic mechanisms, largely through the liver (Wijmenga and Klomp, 2004). Any imbalance of its concentrations, including deficiency and overload, can lead to severe pathological situations, like Wilson’s disease, Menkes disease (Bhattacharya and Thankappan, 2022; Means et al., 2022), and also relevant to cancer. Most importantly, copper acts as a key regulator of some critical signaling pathways to drive biological functions (Grubman and White, 2014). Increasing evidence shows copper was essential for tumorigenesis and tumor progression (Finney et al., 2009; Gupte and Mumper, 2009; Atakul et al., 2020), while excess copper may result in cancer cell cuproptosis (Tang et al., 2022; Tsvetkov et al., 2022). Cuproptosis is a newly recognized cell death form characterized by aggregation of the lipoylated protein, and loss of Fe-S cluster-containing proteins caused by copper overload, finally leading to proteotoxic stress and cell death, distinct from already recognized regulated cell death (Tsvetkov et al., 2022). The report recently published in *Science* identified ten potential CRGs, including *FDX1*, *LIAS*, *LIPT1*, *DLD*, *DLAT*, *PDHA1*, *PDHB*, *MTF1*, *GLS*, *CDKN2A*, and *FDX1* was considered the most important regulator in this cell death process (Tsvetkov et al., 2022). Previous research finds other types of cell death forms play key roles in cancer development and progression and can be targeted for cancer therapies (Su et al., 2015; Liang et al., 2019; Carneiro and El-Deiry, 2020; Zhang et al., 2020; Tan et al., 2021). Recently, increasingly studies identify cuproptosis-related lncRNA as a novel prognostic signature of numerous cancers, such as bladder cancer, cervical cancer, colon cancer (Bai et al., 2022; Wang and Xu, 2022; Xu et al., 2022). However, there is few researches focus on the role of cuproptosis in HCC. Therefore, we combined pan-analysis of CRGs and experimental validation in HCC to confirm the role of CRGs and also constructed a cuproptosis-related prognostic signature in HCC.

Firstly, we comprehensively explored the expression status of CRGs in pan-cancer and its prognostic values in clinical. Our results indicated that CRGs were differentially expressed between cancers and normal tissues and has predictive value for patients’ prognosis; these findings are consistent with previous research (Ji et al., 2018; Zhang et al., 2019; Luan et al., 2021; Zhang et al., 2021). Then, we assessed the expression signature and prognostic values of these ten genes in HCC, most genes had significantly higher expression in HCC compared to normal samples, and different expression of CRGs led to distinct OS periods, which means CRGs was closely correlated to HCC and had the potential for serving as prognostic biomarker of HCC. The expression level of eight prognosis-related CRGs in HCC cell lines was also validated by RT-PCR. Based on the result of RT-PCR, high expression level of *DLAT* and *LIAS* were found in high metastatic HCC cell, while the low metastatic cell had relatively low expression of *DLAT*. So, we chose *DLAT* and *LIAS* for further validation. Protein extracted from surgical specimens was used to perform WB, and the result indicated the expression of DLAT may be correlated to the progression of HCC. Upregulating the expression of *DLAT* in PLC/PRF/5 significantly promoted migration and invasion of HCC cells, while silencing of *DLAT* in MHCC97H exhibits the opposite role. IHC staining of tissue array from HCC cohort revealed that the protein level of *DLAT* was higher in HCC sample than in adjacent non-tumor tissues. Clinically, high expression of DLAT was correlated to shorten OS period and a high recurrence rate after the operation; DLAT was also positively related to microvascular invasion, poor differentiation and advanced TNM stage. *DLAT* also played roles in other types of cancer, Chen et al. (2022) reported that upregulated *DLAT* enhance the malignancy of NSCLC cells, and positively correlated with tumor size and poor prognosis of NSCLC patients. In our study, we identified the expression profiles and prognostic values of CRGs in HCC. Through analyzing the expression levels of CRGs in HCC cell lines and clinical specimens, we found *DLAT* was the most upregulated gene. Furthermore, DLAT showed high expression status in our HCC cohort and closely correlated to dismal outcomes of HCC patients. *In vitro* assays also indicated that *DLAT* drives the metastasis phenotype of HCC cells. These results suggested CRGs, especially *DLAT*, may play a part in the disseminate process of HCC.

Next, lncRNA-mRNA co-expression network was constructed, and a total of 221 lncRNAs were recognized as CRLs. Among these correlated lncRNAs, 29 lncRNAs were recognized as prognosis-related lncRNAs *via* differentially expressed analysis and Univariate Cox analysis. Next, four lncRNAs were screened by LASSO and Cox regression analysis to develop a cuproptosis-related predictive model, including AC011476.3, AC026412.3, NRAV, MKLN1-AS. NRAV, also known as LOC100506668, its expression level in humans was downregulated in virus infection situation. Jing, etc. found it plays key roles in antiviral response by negatively modulating the transcription level of various interferon-stimulated genes (Ouyang et al., 2014). Previous bioinformatic analysis showed lncRNA NRAV involved in HCC (Xu et al., 2021), Endometrial cancer (Wang et al., 2021b), and lower-grade glioma (Maimaiti et al., 2021). Intriguingly, NRAV has been reported to be linked to HCC through multiple cell death forms, including ferroptosis (Chen et al., 2021b), and pyroptosis (Wu et al., 2021). MKLN1-AS also has roles in HCC progression. Guo, etc. reported that Sex-Determining Region Y-Box 9 (*SOX9*) promotes HCC proliferation and EMT *via* regulated MKLN1-AS expression (Guo et al., 2022). Here, we found NRAV and MKLN1-AS also linked to cuproptosis in HCC. Zhang et al. (2022c) have constructed a CRLs signature previously, which have good performances in predicting the outcomes of HCC patients. However, our prognostic model contained only four prognosis-related lncRNAs, which were totally different from the six lncRNAs used by Zhang et al. The ROC curves indicated that our model also exhibited good predictive effects in HCC patient’s prognosis. Furthermore, using RT-PCR, we demonstrated that AC026412.3, MKLN1-AS, NRAV were highly expressed in HCC with disseminate compared to HCC without disseminate. These findings suggested these three lncRNAs may drive HCC metastasis and thus cause the dismal outcomes of HCC patients. Combined bioinformatic analyses and experimental validation made our model more credible.

We constructed a novel risk model containing these four CR-lncRNAs and validated its predictive abilities. Specifically, CRLs related prognostic model was easier to use than other prognostic signatures because it just contained four parameters. Most importantly, our CRLs risk model performs well in predicting HCC patients’ prognosis and can act as the independent risk factor of OS. We also made a comparison among CRLs-related signatures and other cell death-related models; the results indicated our model was better. Nomogram was also established consisting of five factors, including gender, age, grade, stage, and risk, and it has good performance in predicting individual patients’ survival rate at 1, 3, 5 years.

Subsequently, we explore the ICP genes expression in these two groups. The results showed all ICPs expression were significantly higher in high-risk group, which indicated that the high-risk group might be more sensitive to immune checkpoint blockade than low-risk. This discovery was consistent with the published research that copper supplementation upregulated the level of programmed cell death 1 ligand 1 (*PD-L1*) in tumors and thus reduced antitumor immunity (Voli et al., 2020). These observations provide us a thought that CRLs signature can predict the efficacy of immunotherapies.

GSEA was applied to investigate the different biological processes between the high and low risk group. The results showed the biological functions enriched in the high-risk group were mostly immune-related, like cytokine-cytokine receptor interaction, B cell activation, and adaptive immune response. Whereas metabolism-related processes were significantly enriched in the low-risk group, including lipid oxidation and fatty acid catabolic process. The above findings provided explicit perspectives on the difference between distinct CRLs prognostic signatures, which has the potential for expanding therapeutic strategies.

Though we performed a comprehensive analysis of cuproptosis-related genes, there remain several limitations. First, we just preliminary explore the functions of CRGs in HCC, the profound knowledge about the relationship between CRGs and HCC metastasis needs to be investigated by further experiments. Second, our CRLs prognostic signature was established and validated based on the TCGA dataset; we lack patients’ follow-up data from the real world to verify the efficacy of the risk model. Finally, the mechanisms of CRGs involved in the progression of HCC remain unclear.

## 5 Conclusion

The prognostic value of CRGs in HCC was verified by bioinformatic analysis and experimental validation. We developed a novel CR-lncRNA risk model, which has favorable performance for predicting OS in HCC patients. A simplified nomogram consisting of gender, age, grade, stage, and risk was also constructed, it can be the clinical tool for forecasting the outcome of individual patients. CRLs prognostic signature also can reflect the sensitivity of HCC patients to immunotherapy and chemotherapy agents, which has crucial roles in improving the effectiveness of treatments, reducing the adverse effects, and saving medical resources.

## Data Availability

The datasets presented in this study can be found in online repositories. The names of the repository/repositories and accession number(s) can be found in the article/Supplementary Material.
