# Supplementary material for: An integrative analysis revealing cuproptosis-related lncRNAs signature as a novel prognostic biomarker in hepatocellular carcinoma
Source: Front Genet. 2023 Feb 10;14:1056000. doi: 10.3389/fgene.2023.1056000 (PMC9950118; doi:10.3389/fgene.2023.1056000)
Supplement: Supplementary file 1 [file DataSheet1.docx]

Supplementary Material

# Methods

**Cell Culture**

Six HCC cell lines were used in this study, including, Huh6 was purchased from the Institute of Biochemistry and Cell Biology, Chinese Academy of Science, China. PLC/PRF/5, SNU398 were purchased from the American Type Culture Collection. HCCLM3, HCCLM6, and MHCC97H were kindly provided by Dr. Tang ZY (Liver cancer Institute, Zhongshan Hospital, Fudan University, Shanghai, China). And cell lines confirmed no mycoplasma contamination. Cells were cultured in Dulbecco’s modified Eagle’s medium (DMEM) supplemented with 10% FBS (Gibco, United States) and 100 U/ml penicillin-streptomycin (Gibco, United States) and placed in 5% CO_2_ cell incubator at 37°C.

**Lentivirus transduction**

Lentivirus carrying sh-DLAT, OE-DLAT and lentivirus carrying sh-NC and OE-NC was obtained from Shanghai GeneChem Co., Ltd., (China). Three short hairpin RNAs (shRNAs) sequences were presented in **Supplementary Table S1**. Lentiviral infection of target cells was performed in cell culture media with 5 μg/ml polybrene (Sigma H9268). Seventy-two hours after infection, cells were selected for 2 weeks using 2.5 μg/ml puromycin (OriGene). Selected pools of cells were used for the following experiments. Puromycin-resistant cells were amplified for 9 days in medium that contained 2 μg/mL puromycin, and then transferred to medium without puromycin. Stable DLAT-deficient LoVo cells and DLAT-overexpressing SW480 cells were then obtained.

**Real-time qPCR**

The RNeasy Plus Mini Kit (50) kit (Qiagen, Hilden, Germany) was used to extract total RNA, which was then reverse transcribed with the Advantage RT-for-PCR Kit (Qiagen) in accordance with the manufacturer’s protocols. The target sequence was amplified with real-time PCR with the SYBR Green PCR Kit (Qiagen). The cycling parameters used were 95 °C for 15 s, 55-60 °C for 15 s, and 72 °C for 15 s for 45 cycles. Melting curve analyses were performed, and Ct values were determined during the exponential amplification phase of real-time PCR. SDS 1.9.1 software (Applied Biosystems, Massachusetts, USA) was used to evaluate amplification plots. The 2^–ΔΔCt^ method was used to determine relative fold changes in target gene expression in cell lines, which was normalized to expression levels in corresponding control cells (defined as 1.0). The equation used was 2^–ΔΔCt^ (ΔCt = ΔCt^target^ – ΔCt^GAPDH^; ΔΔCt = ΔCt^expressing vector^ – ΔCt^control vector^). When calculating relative expression levels in surgically extracted CRC samples, relative fold changes in target gene expression were normalized to expression values in normal colon epithelial tissues (defined as 1.0) using the following equation: 2 ^–ΔΔCt^ (ΔΔCt = ΔCt^tumor^ – ΔCt^nontumor^). All experiments were performed in duplicate. **Supplementary Table S2** lists all sequences of all primers used in this study.

**Western blot analyses**

Proteins from lysed cells were fractionated by SDS-PAGE and transferred to nitrocellulose membranes. Nonspecific binding sites were blocked with 5% milk in TBST (120 mM Tris–HCl (pH 7.4), 150 mM NaCl, and 0.05% Tween 20) for 1 hour at room temperature. Blots were incubated with a specific antibody overnight at 4 °C. Western blotting of β-actin on the same membrane was used as a loading control. The membranes were incubated with primary antibodies overnight at 4 °C. The membranes were then washed with PBS 3 times and incubated with an HRP-conjugated secondary antibody. Proteins were visualized using a Immobilon^TM^ Western Chemiluminescent HRP substrate(Millipore, USA). Antibodies used in this study were as follow DLAT (ab110332, Abcam), LIAS (11577-1-AP, Proteintech), β-Actin (66009-1-lg, Proteintech).

**Patients and follow-up**

This study was approved by the Ethics Committee of Tongji hospital of Tongji Medical College. All patients provided full consent for the study. HCC Cohort included 126 adult patients with HCC who underwent curative resection between 2006 and 2008 at the Tongji Hospital of Tongji Medical College (Wuhan, China). A preoperative clinical diagnosis of HCC was based on the diagnostic criteria of the American Association for the Study of Liver Diseases. The inclusion criteria were as follows: (a) distinctive pathologic diagnosis; (b) no preoperative anticancer treatment or distant metastases; (c) curative liver resection; and (d) complete clinicopathologic and follow-up data. The differentiation statuses were graded according to the method of Edmondson and Steine. The TNM classification for HCC was based on The American Joint Committee on Cancer/International Union Against Cancer staging system (6th edition, 2002). Follow-up data were summarized at the end of December 2016 (range 4-96 months). The patients were evaluated every 2-3 months during the first 2 years and every 3-6 months thereafter. All follow-up examinations were performed by physicians who were blinded to the study. During each check-up, the patients were monitored for tumor recurrence by measuring the serum AFP level and by performing abdominal ultrasound examinations. A computed tomography and/or magnetic resonance imaging examination was performed every 3-6 months, together with a chest radiographic examination. The diagnostic criteria for HCC recurrence were the same as the preoperative criteria. The time to recurrence and overall survival were the primary endpoints. The time to recurrence was calculated from the date of resection to the date of a diagnosis with tumor recurrence. The overall survival was calculated from the date of resection to the date of death or of the last follow-up.

In addition, 15 pairs of fresh metastatic and matched primary HCC tissues were collected after surgical resection and were used for further investigations.

**Immunohistochemistry**

Immunohistochemistry was performed on 4-μm-thick, routinely processed paraffin-embedded sections. Briefly, after baking on a panel at 60 °C for an hour, the tissue sections were deparaffinized with xylene and rehydrated through gradient ethanol immersion. Endogenous peroxidase activity was quenched by 3% (vol/vol) hydrogen peroxide in methanol for 12 min, followed by three 3-min washes with phosphate-buffered saline (PBS). Then the slides were immersed in 0.01 mol/L citrate buffer solution (pH 6.0) and placed in a microwave oven for 30 min. After washing in PBS (pH 7.4, 0.01 mol/L), the sections were incubated in a moist chamber at 4 °C overnight with the primary antibody diluted in PBS containing 1% (wt/vol) bovine serum albumin. Negative controls were performed by replacing the primary antibody with preimmune mouse serum. After three 5 min washes with PBS, the sections were treated with a peroxidase-conjugated second antibody (Santa Cruz) for 30 min at room temperature, followed by additional three 5 min washes with PBS. Reaction product was visualized with diaminobenzidine for 2 min. Images were obtained under a light microscope (Olympus, Japan) equipped with a DP70 digital camera.

Analyses were performed by two independent observers who were blinded to the clinical outcome. The immunostaining intensity was scored on a scale of 0 to 3: 0 (negative), 1 (weak), 2 (medium) or 3 (strong). The percentage of positive cells was evaluated on a scale of 0 to 4: 0 (negative), 1 (1%-25%), 2 (26%-50%), 3 (51%-75%), or 4 (76%-100%). The final immuno-activity scores were calculated by multiplying the above two scores, resulting an overall scores which range from 0~12. Each case was ultimately considered “negative” if the final score ranges from 0~3, and “positive” if the final score ranges from 4~12.

***In vitro* migration and invasion assays**

The invasive and migratory capabilities of each cell line were assessed with an 8-µm pore, 24-well Transwell plate (Corning, USA). For invasion assays, chamber inserts were first coated with 60 μL of Matrigel (Corning, 200 mg/mL) and left to dry overnight under sterile conditions. The next day, the uppermost chamber was plated at a cell density of 1×10^5^. For cell migration assays, the upper chamber, which was lined with a noncoated membrane, was plated with cells at a density of 5×10^4^. Each assay was repeated thrice, and three different inserts were used to obtain a mean cell number in five fields per membrane.

**Quantification and Statistical Analysis**

**Statistical Analysis**

R software (4.1.2) was employed to carry out bioinformatic statistical analysis and graph plotting. The Wilcox test was applied to analyze the differences between the two groups. Kaplan-Meier survival analysis and univariate Cox regression model were used to determine the correlation between CRGs and prognosis. Survival analysis was performed by the Kaplan-Meier method, and the log-rank test was applied to calculate the p-value of the difference. Spearman correlation analysis and distance correlation analysis were performed in the correlation test. “pheatmap” package was used to draw all the heatmaps. The quantitative data were compared between groups using the Student's t-test. Categorical data were analyzed using the Fisher’s exact test. The cumulative recurrence and survival rates were determined using the Kaplan-Meier method and log-rank test. A value of p < 0.05 was considered to be significant. The analyses were performed using the SPSS software (version 26.0).

# Supplementary Figures


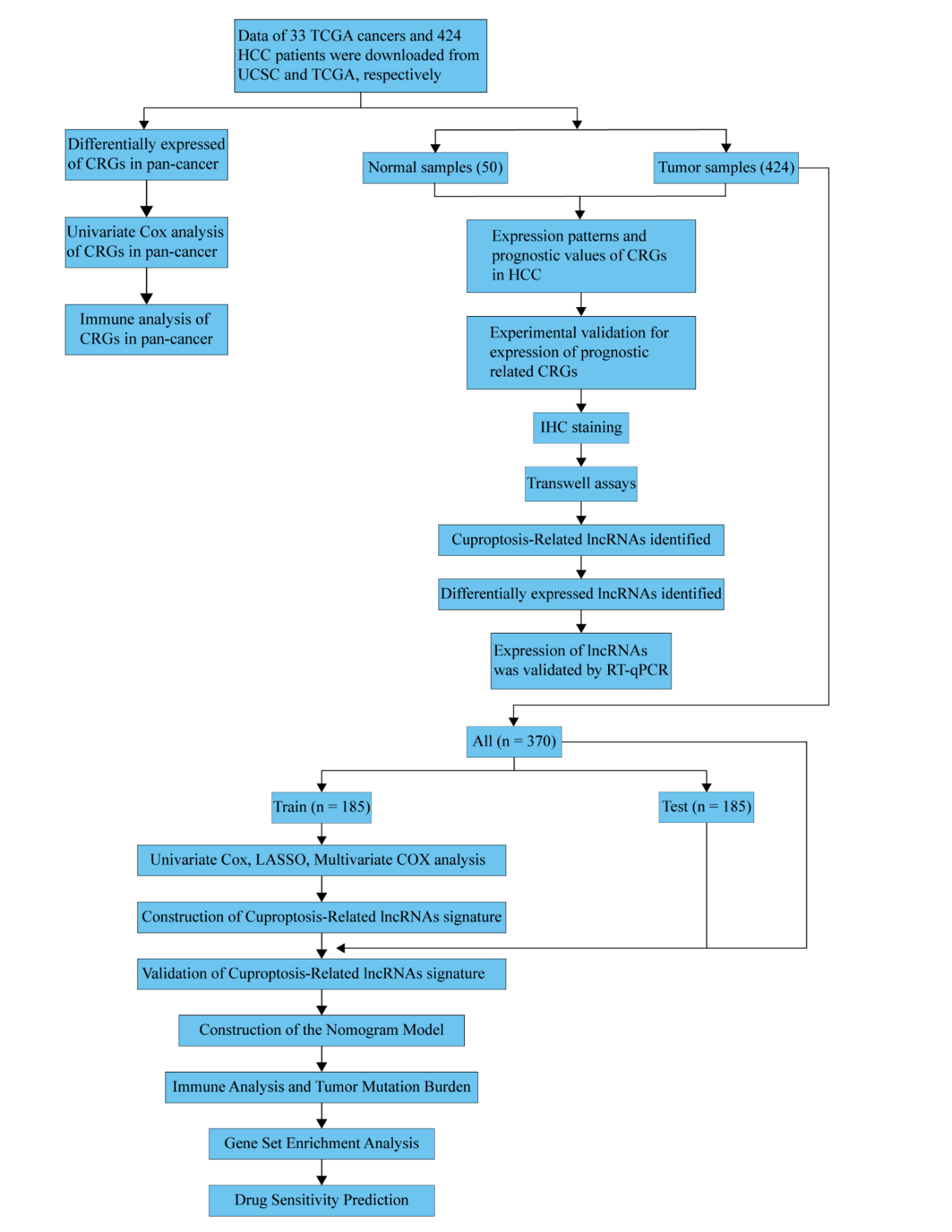


**Supplementary Figure 1.** The flow chart for constructing cuproptosis-related lncRNAs signature.


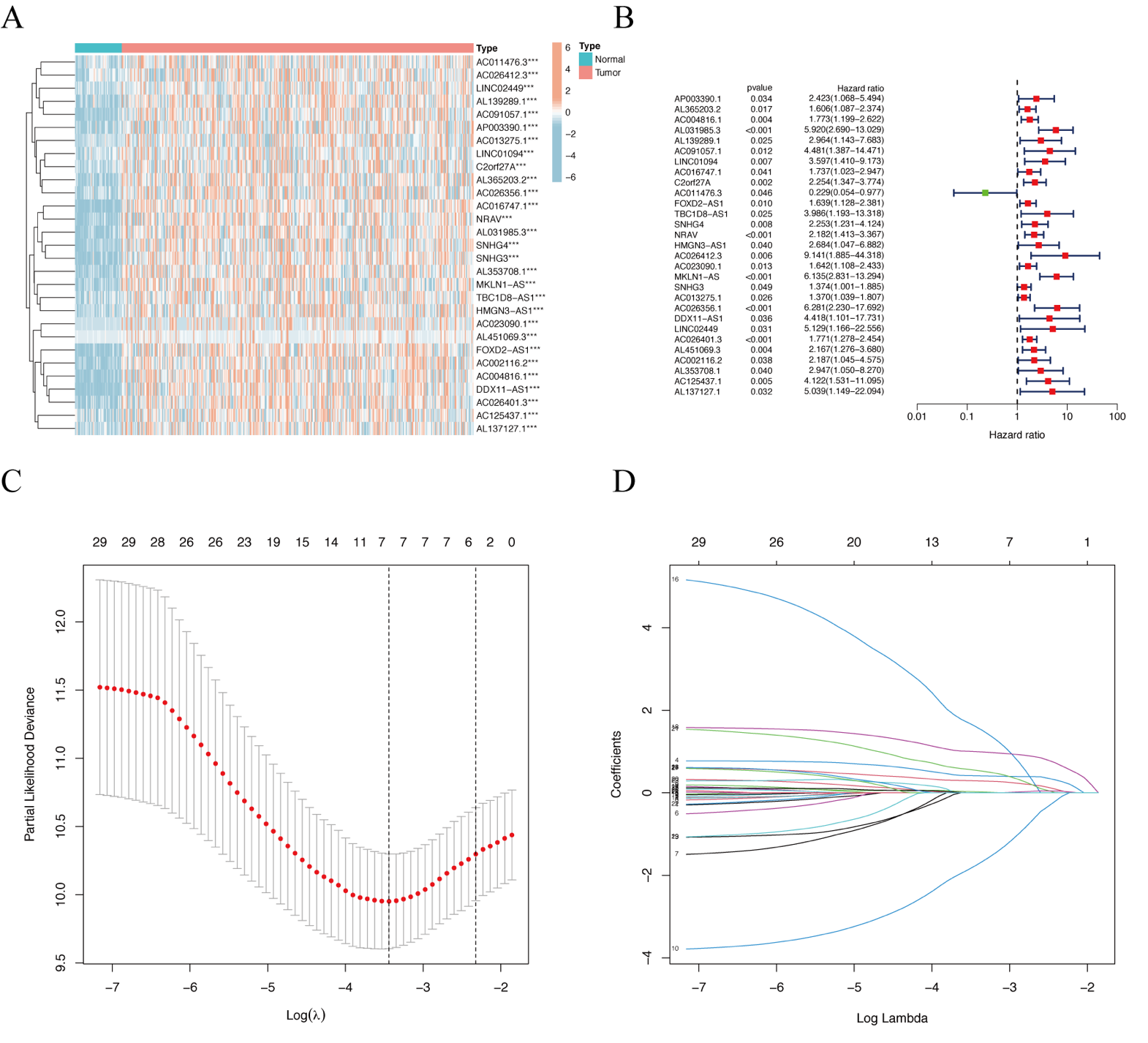


**Supplementary Figure 2.** Construction of cuproptosis-related lncRNAs signature. **(A)** Heat map of differentially expressed cuproptosis-related lncRNAs in normal and HCC cancer. **(B)** The Forest plot shows the prognostic value of cuproptosis-related lncRNAs. **(C - D)** LASSO COX regression analysis.


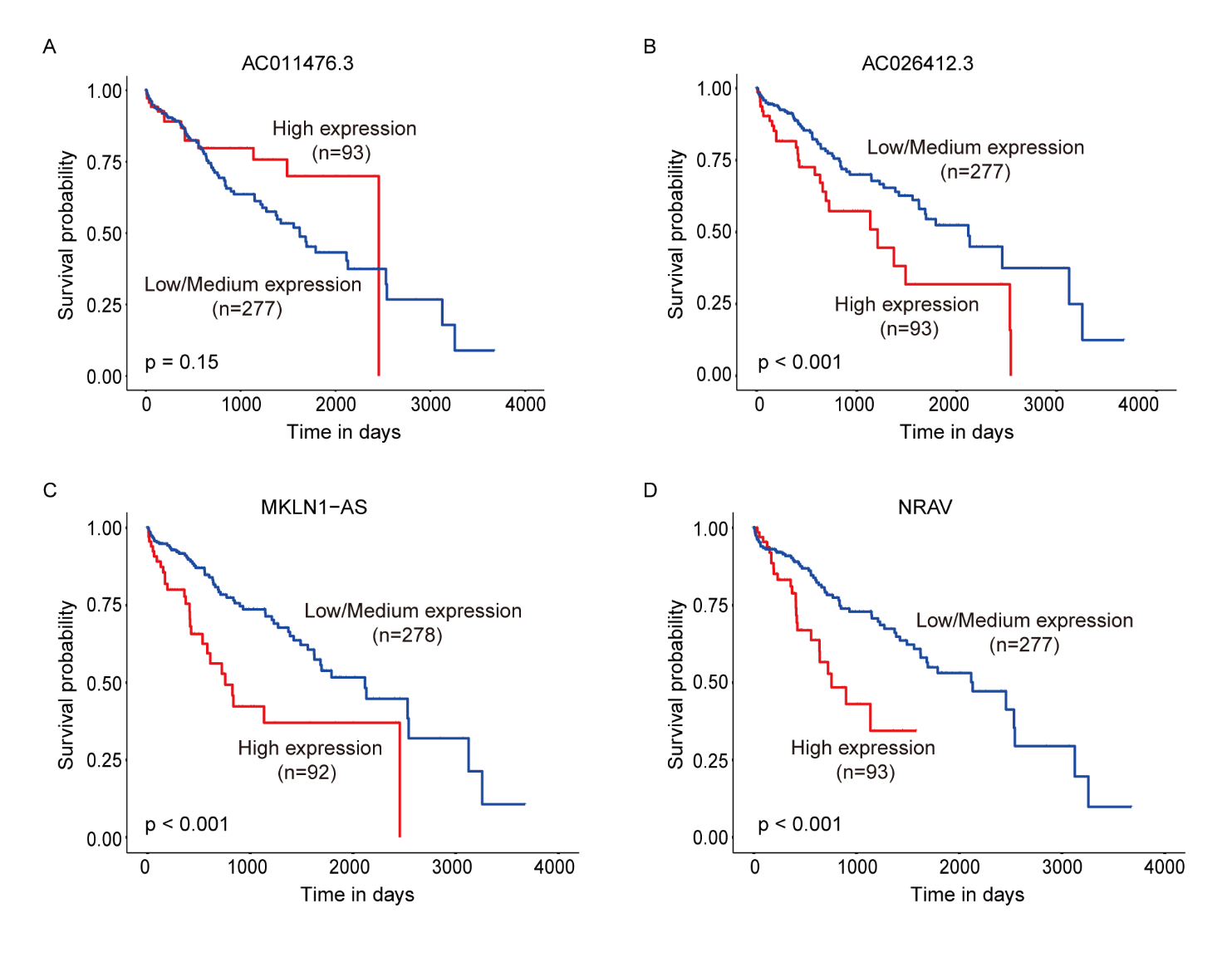


**Supplementary Figure 3.** The correlation between lncRNA and overall survival periods was explored by UALCAN online tool. **(A)** AC011476.3. **(B)** AC026412.3. **(C)** MKLN1-AS. **(D)** NRAV.


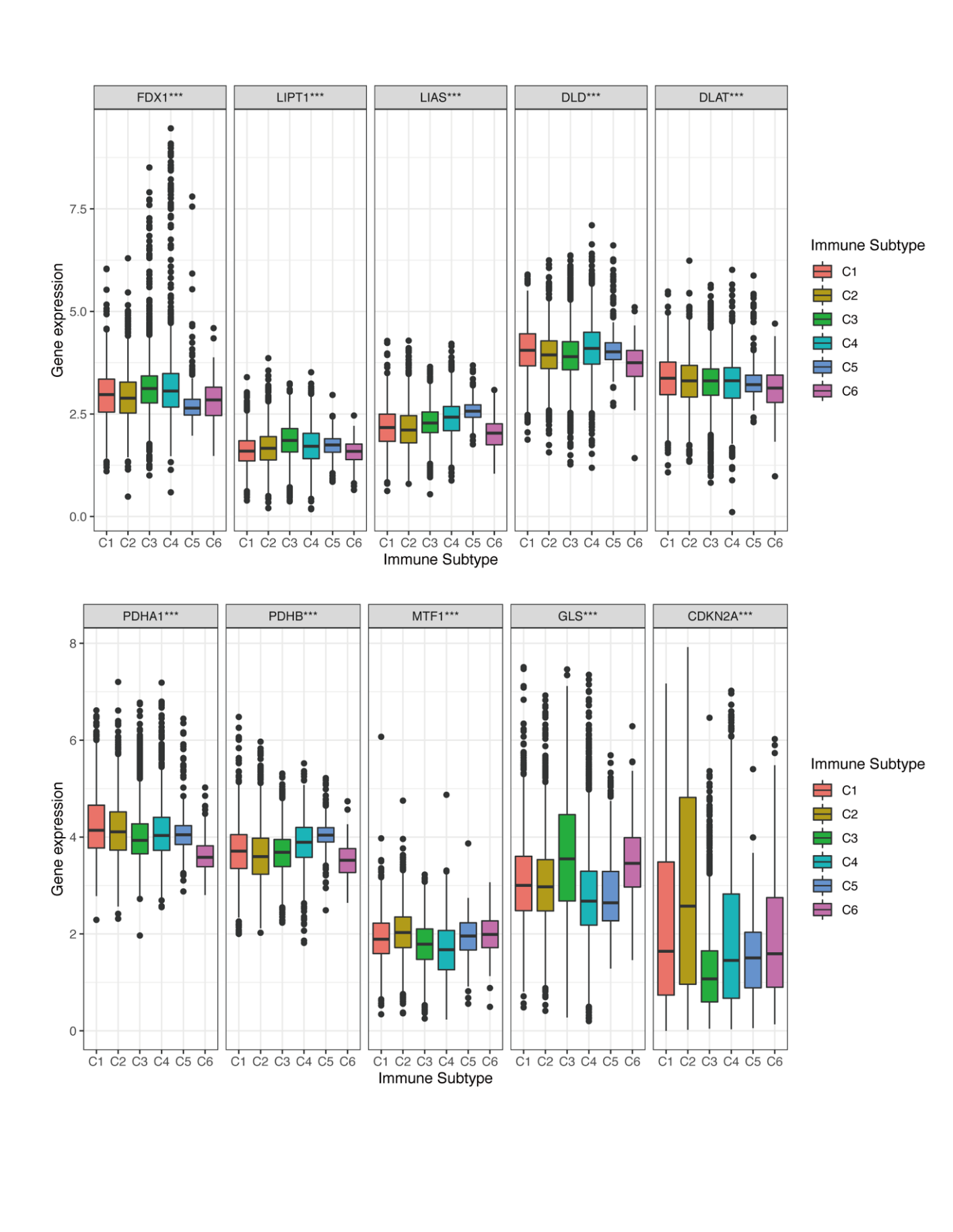


**Supplementary Figure 4.** The expression level of 10 CRGs in different immune subtypes (C1-C6).


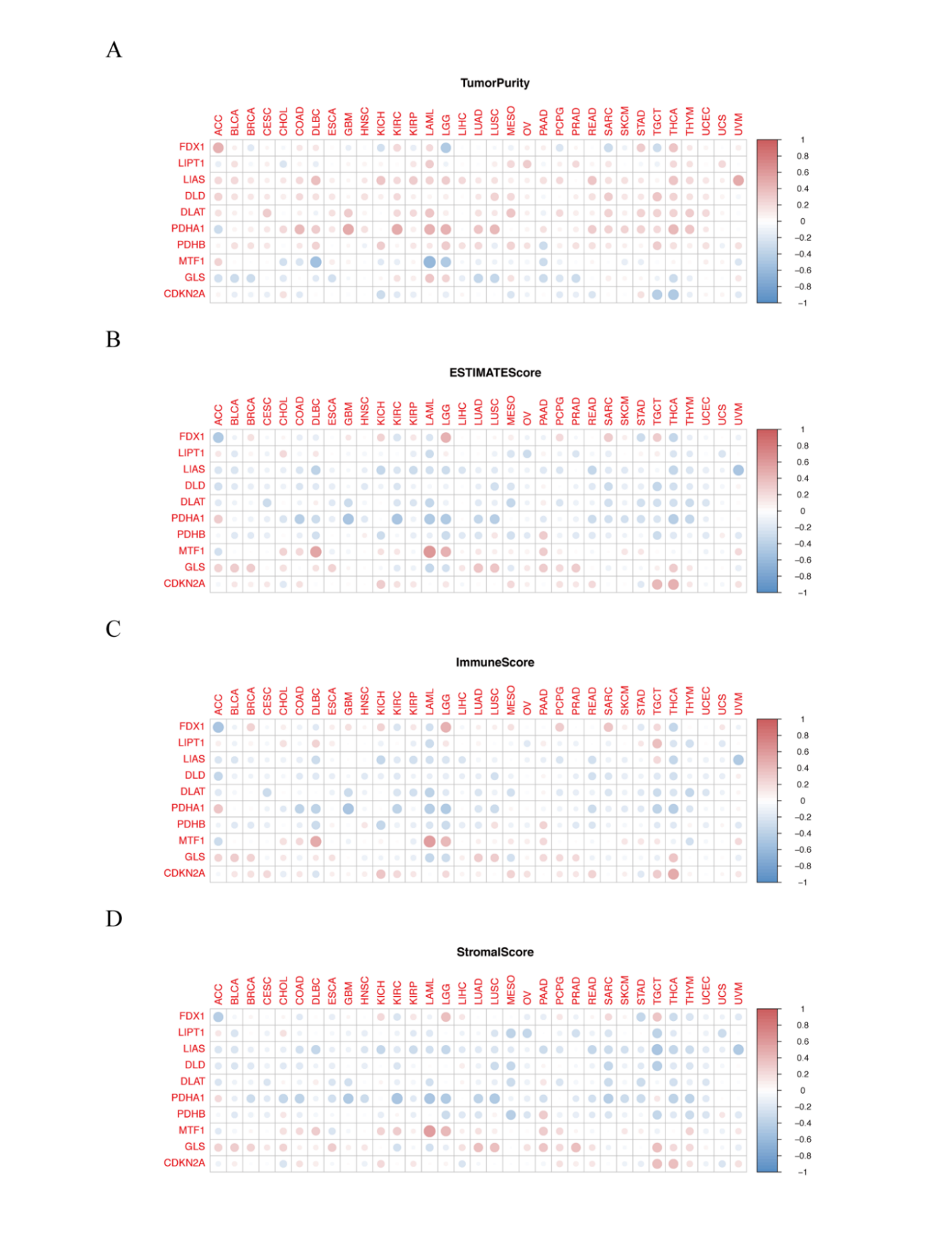


**Supplementary Figure 5.** The correlation of 10 CRGs and immune scores in TCGA cancer. **(A)** Tumor purity. **(B)** ESTIMATEScore. **(C)** ImmuneScore. **(D)** StromalScore.


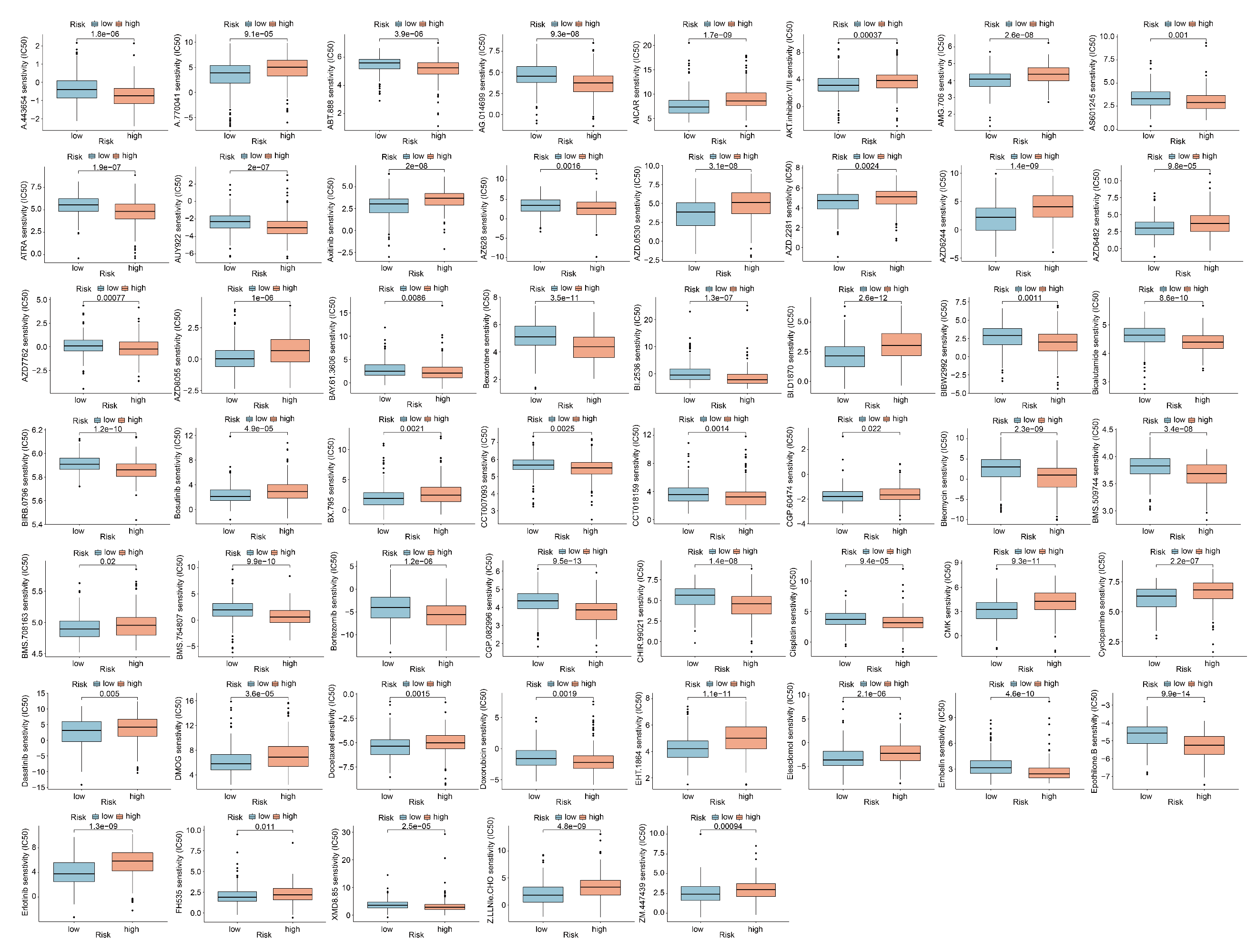


**Supplementary Figure 6. Correlation between distinct risk groups and drugs sensitivity.**

**
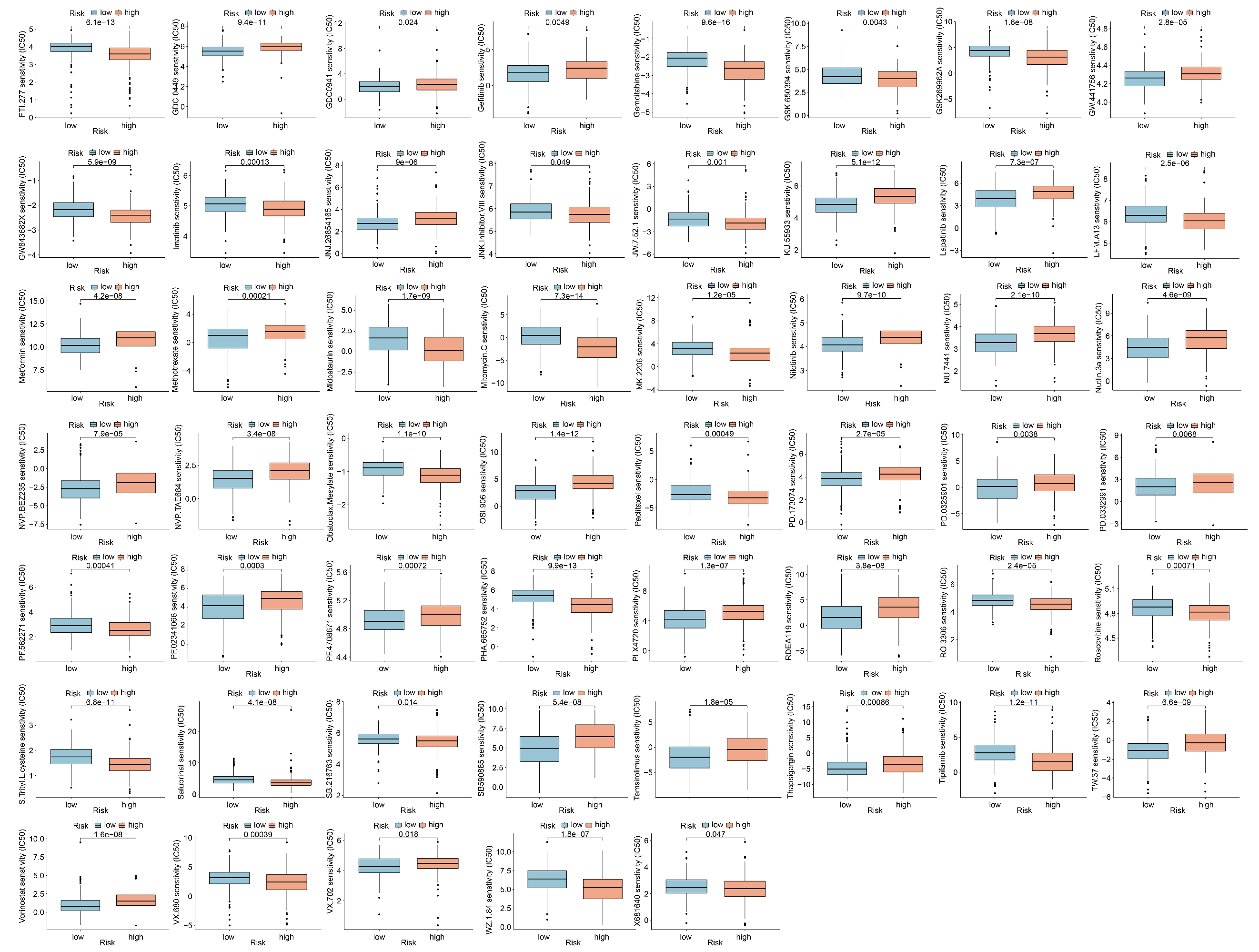
**

**Supplementary Figure 7. Correlation between distinct risk groups and drugs sensitivity (continued).**

# Supplementary Tables

**Supplementary Table S1. Knockdown shRNA sequences used in this study**

TRC number Sequence

DLAT

shRNA-1 CCGGCCATACCTCATTATTACCTTTCTCGAGAAAGGTAATAATGAGGTATGGTTTTTG

shRNA-2 CCGGCCACTCTGTATCATTGTAGAACTCGAGTTCTACAATGATACAGAGTGGTTTTTG

shRNA-3 CCGGGCAGAGGTTGAAACTGATAAACTCGAGTTTATCAGTTTCAACCTCTGCTTTTTG

**Supplementary Table S2. Primer sequences used in the study**

| Primer name | Primer sequences |  |
| --- | --- | --- |
| Primers for real-time PCR: |  |  |
| β-actin sense: | 5'- CTCCATCCTGGCCTCGCTGT -3' |  |
| β-actin antisense: | 5'- GCTGTCACCTTCACCGTTCC -3' |  |
| CDKN2A sense: | 5’- ACTCTCACCCGACCCGT -3’ |  |
| CDKN2A antisense: | 5’- CATCTATGCGGGCATGGTTACT -3’ |  |
| DLAT sense: | 5’- ACCAAAGCAAGAGAGGGTAAACT -3’ |  |
| DLAT antisense: | 5’- AGACATCATGCTAGCCACATCAA -3’ |  |
| FDX1 sense: | 5’- TCTTTGGAGTCTCTCGCGG-3’ |  |
| FDX1 antisense: | 5’-ACTGTTATTTTATCTTCTGAGCTGC -3’ |  |
| GLS sense | 5’- ACCAAAGTTCCCTTCTGTCTTCA-3’ |  |
| GLS antisense | 5’-TGAAGTCACAACAATTGCTCCAG-3’ |  |
| LIAS sense | 5’-CCAGTGAGCCCTACAATACTGC-3’ |  |
| LIAS antisense | 5’-AGGATCACGAACCTTACTTTCCT-3’ |  |
| LIPT1 sense | 5’-GCTCTGAATGCTGTCCAACCC-3’ |  |
| LIPT1 antisense | 5’-GCAATGGTGATAGGCAGTAGTCC-3’ |  |
| MTF1 sense | 5’-CGAAGGAGAAGCCATTTGAG-3’ |  |
| MTF1 antisense | 5’-TGGCTTTTCCCCTGTATGAG-3’ |  |
| PDHB sense | 5’-GAAGAGGCGCTTTCACTGGA-3’ |  |
| PDHB antisense | 5’-CAGCCCTCGACTAACCTTGT-3’ |  |
| NRAV sense | 5'- GGAGTTGATGCCTCCGAACA-3' |  |
| NRAV antisense | 5'-ATGACCGGAGCTGAAAGGTG-3' |  |
| MKLN1-AS sense | 5'-GATAACCGTCCAGGAATGAGAG-3' |  |
| MKLN1-AS antisense | 5'-CCCAGCCACCAAACAAATAAA-3' |  |
| AC011476.3 sense | 5'-GGCAGTCAAGATGGAGGACC-3' |  |
| AC011476.3 antisense | 5'- AGCACTTCCTGTATCCACCG-3' |  |
| AC026412.3 sense | 5'- CCTAATTCCAGGGCGGATGG -3' |  |
| AC026412.3 antisense | 5'- TCCCAAAGTCACGGCCTTC -3' |  |
